# Shannon Entropy in LS-Coupled Configuration Space for Ni-like Isoelectronic Sequence

**DOI:** 10.3390/e24020267

**Published:** 2022-02-12

**Authors:** Jian-Jie Wan, Jie Gu, Jiao Li, Na Guo

**Affiliations:** College of Physics and Electronic Engineering, Northwest Normal University, Lanzhou 730070, China; guj102470@163.com (J.G.); lj2978567745@163.com (J.L.); guona020415@163.com (N.G.)

**Keywords:** Shannon entropy, LS-coupled configuration space, LS-jj transformation, unique notation

## Abstract

The Shannon entropy in an LS-coupled configuration space has been calculated through a transformation from that in a jj-coupled configuration space for a Ni-like isoelectronic sequence. The sudden change of Shannon entropy, information exchange, eigenlevel anticrossing, and strong configuration interaction have been presented for adjacent levels. It is shown that eigenlevel anticrossing is a sufficient and necessary condition for the sudden change of Shannon entropy, and both of them are a sufficient condition for information exchange, which is the same as the case of the jj-coupled configuration space. It is found that the structure of sudden change from jj-coupled into LS-coupled configuration spaces through the LS-jj transformation is invariant for Shannon entropy along the isoelectronic sequence. What is more, in an LS-coupled configuration space, there are a large number of information exchanges between energy levels whether with or without strong configuration interaction, and most of the ground and single excited states of Ni-like ions are more suitable to be described by a jj-coupled or other configuration basis set instead of an LS-coupled configuration basis set according to the configuration mixing coefficients and their Shannon entropy. In this sense, Shannon entropy can also be used to measure the applicability of a configuration basis set or the purity of atomic state functions in different coupling schemes.

## 1. Introduction

Shannon information entropy [1] has been used to describe a large variety of physical concepts nowadays and to elucidate the physical and chemical properties of atomic and molecular systems. To our knowledge, over the past few decades, there have been two main kinds of theoretical work related to Shannon entropy in the field of atomic and molecular physics. The first is the theoretical research related to the localization or delocalization (nonlocality) of electron clouds in position space and momentum space (e.g., [2,3,4,5,6,7,8,9,10,11,12]). The second kind of theoretical work is to describe the complexity of electron clouds in position space and momentum space (e.g., [13,14,15,16,17,18]). As far as we know, in atomic and nuclear physics, Shannon entropy has also been used to study the quantum chaotic system [19,20,21] by using the configuration interaction method to analyze the spectrum and the eigenstates of the complex atom and heavy nuclei, in which the wavefunction of the excited states are chaotic superpositions of hundreds or thousands of principal basis states [22,23]. Recently, in our previous work [24,25], we have found the following in a jj-coupled configuration space: (1) The sudden change of Shannon entropy is a sufficient and necessary condition for the eigenlevel anticrossing in a given configuration space whether the total angular momentum and parity $J^{P}$ of the adjacent levels is the same or not, with the help of which it is easy to determine the position of eigenlevel anticrossing; the transition probabilities can be changed dramatically around the anticrossing of eigenlevel, and strongly induced transition in an external electromagnetic field can take place; (2) The sudden change of Shannon entropy is a sufficient condition for information exchanges whether $J^{P}$ of the adjacent levels is the same or not; (3) There is no necessary causal relationship between the eigenlevel anticrossing and strong configuration interaction in isoelectronic sequences.

However, we do not know whether the same conclusion exists in an LS-coupled configuration space, in other words, whether the transformation between the different coupling basis sets affects the above conclusion. In addition, there is indeed no discussion about the information in an LS-coupled configuration space, which is expanded by the LS-coupled configuration basis set that could be transformed into from the jj-coupled configuration basis set, although the Shannon entropy was discussed in the jj-coupled configuration space for a Ni-like isoelectronic sequence [25] in detail. Therefore, it is necessary to check whether there is any difference between the LS- and jj-coupled configuration space, and the present work is a continuation of our previous work [24,25] in the LS-coupled configuration space. In this paper, discrete Shannon entropy in the LS-coupled configuration space has also been calculated to measure information on the atomic states. In Section 2, we provide a brief description of the Shannon entropy in jj- and LS-coupled configuration spaces and the unique algorithm, which can be used to label a level by a configuration state function (CSF) uniquely in a given configuration space, as proposed by Gaigalas et al. [26]. In Section 3, the Shannon entropies have been presented for the ground and single excited states in the LS-coupled configuration space along with the Ni-like isoelectronic sequence. Compared with the Shannon entropy in the jj-coupled configuration space, the Shannon entropy of the corresponding energy level in the LS-coupled configuration space is checked in detail. Then, the relationship between the sudden change of Shannon entropy, information exchange, eigenlevel anticrossing, and strong configuration interaction has been discussed based on the calculated energy levels, configuration mixing coefficients, and Shannon entropies. Finally, some concluding remarks and outlook are summarized in Section 4.

## 2. Theoretical Considerations

In our previous work [24,25], the atomic state wavefunction was expressed by an expansion of the jj-coupled configuration basis set, which is obtained by using the relativistic configuration interaction (RCI) with relativistic one-electron orbitals and multiconfiguration Dirac–Hartree–Fock (MCDHF) methods with the relativistic electron orbitals generated by the self-consistent field procedure [27,28,29,30,31].

(1) $$\begin{array}{c} |\Psi_{r}\left(J^{P}\right)\rangle =\sum_{s=1}^{n_{c}}C_{rs}^{\left(jj\right)}|\Gamma_{s}\left(J^{P}\right)\rangle ,r=1,2,\ldots ,n_{c}, \end{array}$$

where $|\Psi_{r}\left(J^{P}\right)\rangle$ is the *r*th atomic state function (ASF) describing the *r*th level. $|\Gamma_{s}\left(J^{P}\right)\rangle ,s=1,2,\ldots ,n_{c}$ are the configuration state functions (CSF) in the jj-coupled configuration space. In this paper, the even and odd parities are described by the superscripts *e* and *o*. $n_{c}$ is the number of the configuration state functions, which describes the size of the configuration space. $C_{rs}^{\left(jj\right)},s=1,2,\ldots ,n_{c}$ are the configuration mixing coefficients for the *r*th atomic state function in the jj-coupled configuration space, and the modulus square $|C_{rs}^{\left(jj\right)}{|}^{2}$ indicates the weight of the *s*th configuration in the *r*th atomic state, yielding the normalization condition for the *r*th atomic state function

(2) $$\begin{array}{c} \sum_{s=1}^{n_{c}}{\left|C_{rs}^{\left(jj\right)}\right|}^{2}=1,r=1,2,\ldots ,n_{c}. \end{array}$$

Usually, when the distribution of configuration weights is localized, the energy level is labeled by the dominant component in an expansion of atomic state function, i.e., the configuration with the largest modulus square, which is usually considered as the notation of energy level, and then, the notation is regarded as the information of a given level. In other words, the more remarkable localization of the distribution of configuration weights is, the more certain the information of the energy level. In this case, the dominant component is just unique notation. However, for many energy levels, especially for highly excited states, there are two or more atomic state functions having the same dominant components to show nonunique labeling for energy levels as mentioned by Gaigalas et al. [26]. So, a new algorithm has been proposed to define the unique notation for all levels in a subspace with the same $J^{P}$. Most simply, for a given configuration space, the CSF with the largest configuration weight is used as the notation for the level. Once a CSF notation is assigned, the corresponding CSF notation is removed from consideration in the determination of the notation of next level. In the present work, reassigned unique notation marked by the symbol ‘*’ is performed for those levels that have the same dominant CSF in a given configuration space with a certain $J^{P}$. In other words, in those figures and tables in Section 3, when the unique notations are just the dominant components, their coefficients are written only in bold font. However, the coefficients are written in bold font and marked by ‘*’ when the unique notations are not the dominant components. In view of this, each level is labeled as the so-called unique notation instead of the dominant component in this paper. It can be seen that the dominant-component labeling can be regarded as a special case of the unique labeling.

In our previous work [24,25], a discrete Shannon entropy had been set up to measure the information in the jj-coupled configuration space because of the properties of configuration mixing coefficients, that is, the configuration weights

(3) $$\begin{array}{c} \rho_{rs}^{\left(jj\right)}\equiv {\left|C_{rs}^{\left(jj\right)}\right|}^{2}\in \left[0,1\right],r,s=1,2,\ldots ,n_{c}, \end{array}$$

yield the normalization condition

(4) $$\begin{array}{c} \sum_{s=1}^{n_{c}}\rho_{rs}^{\left(jj\right)}=1,r=1,2,\ldots ,n_{c}. \end{array}$$

Therefore, the Shannon entropy of the *r*th energy level described by the *r*th atomic state function $|\Psi_{r}\left(J^{P}\right)\rangle$ is defined by

(5) $$\begin{array}{c} S_{\Psi_{r}}^{\left(jj\right)}=-\sum_{s=1}^{n_{c}}\rho_{rs}^{\left(jj\right)}\ln\rho_{rs}^{\left(jj\right)},r=1,2,\ldots ,n_{c}, \end{array}$$

which can be used to indicate the information on a certain energy level in quantity; i.e., the Shannon entropy can measure the uncertainty of the configurations for each certain atomic state in a given jj-coupled configuration space.

As is well known, in atomic spectroscopy, the LS-coupled configuration notation is also applied for classifying the levels of atoms and ions frequently. That is, the atomic state function can also be written as an expansion of configuration basis set in LS-coupled configuration space, i.e.,

(6) $$\begin{array}{c} |\Psi_{r}\left(J^{P}\right)\rangle =\sum_{s=1}^{n_{c}}C_{rs}^{\left(LS\right)}|\Gamma_{s}\left(LSJ^{P}\right)\rangle ,r=1,2,\ldots ,n_{c}, \end{array}$$

Then, the Shannon entropy in LS-coupled configuration space is expressed by

(7) $$\begin{array}{c} S_{\Psi_{r}}^{\left(LS\right)}=-\sum_{s=1}^{n_{c}}\rho_{rs}^{\left(LS\right)}\ln\rho_{rs}^{\left(LS\right)},r=1,2,\ldots ,n_{c}, \end{array}$$

where $\rho_{rs}^{\left(LS\right)}$ is the weight of the *s*th configuration state function $|\Gamma_{s}\left(LSJ^{P}\right)\rangle$ in the *r*th atomic state function $|\Psi_{r}\left(J^{P}\right)\rangle$. In the present work, the configuration mixing coefficients in the jj-coupled configuration space are transformed into those in the LS-coupled configuration space through the method, which was proposed by Gaigalas et al. [26,32,33] and is not necessary to be described in detail in this paper. Therefore, the Shannon entropy in the LS-coupled configuration space can be obtained for the energy levels of ground and single excited states of Ni-like isoelectronic sequence. Likely in [25], the LS-coupled subspaces with even and odd parity for the energy levels of Ni-like ions have been shown in Table 1 and Table 2, respectively, where the closed subshells have been omitted for convenience.

## 3. Results and Discussion

In our previous work [24,25], we calculated the Shannon entropies of the ground and singly excited states in a jj-coupled configuration space for a Ni-like isoelectronic sequence and focused on the relationship between the sudden change of Shannon entropy, eigenlevel anticrossing, information exchange, and strong configuration interaction. In the present work, all figures present the Shannon entropies for the levels in the LS-coupled configuration space, configuration weights and eigenlevel anticrossing for selected levels and the unique notation of each level has also been given in these figures according to the configuration mixing coefficients.

**Figure 1:**
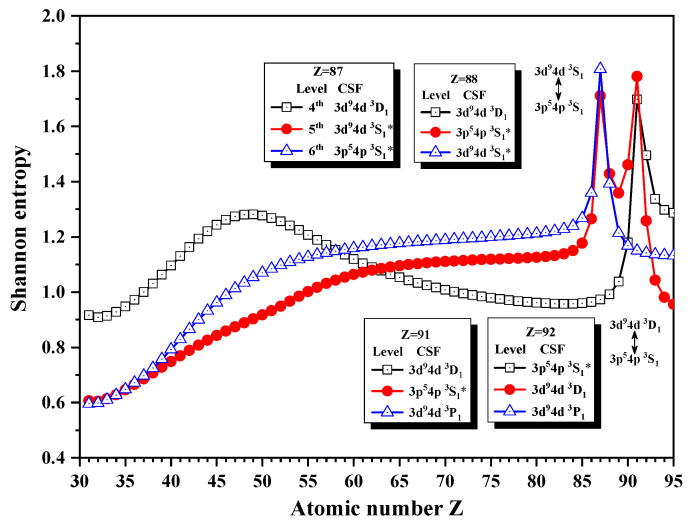
Shannon entropies for the 4th, 5th, and 6th levels in the LS-coupled subspace with $J^{P}=1^{e}$ for Ni-like isoelectronic sequence with Z = 31–95.

**Figure 2:**
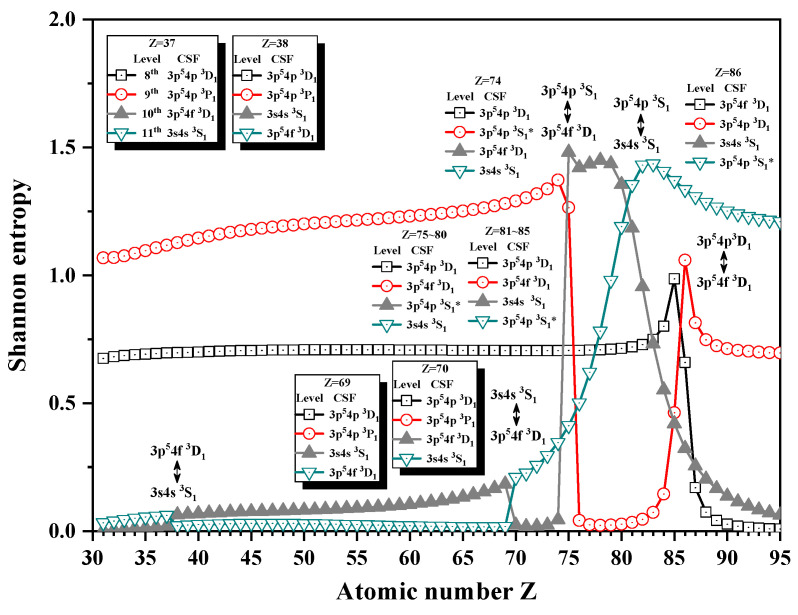
Shannon entropies for the 8th, 9th, 10th, and 11th levels in the LS-coupled subspace with $J^{P}=1^{e}$ for Ni-like isoelectronic sequence with Z = 31–95.

Figure 1 and Figure 2 show the Shannon entropies in an LS-coupled configuration space and the unique notations for seven $J^{P}=1^{e}$ levels of a Ni-like isoelectronic sequence. In Figure 1, the Shannon entropies have maxima at Z = 87 for the 5th and 6th levels, while for the 4th and 5th levels, they have maxima at Z = 91, which is likely in Figure 1 in [25]. According to the configuration mixing coefficients, the information of eigenlevels is exchanged around the position of the peaks. Definitely, the 4th, 5th, and 6th levels are in turn labeled by the so-called unique notations 3d${}^{9}$4d ${}^{3}D_{1}$, 3d${}^{9}$4d ${}^{3}S_{1}*$ and 3p${}^{5}$4p ${}^{3}S_{1}*$ in the configuration subspace with $J^{P}=1^{e}$ for Z = 87. At Z = 88, the unique notations 3d${}^{9}$4d ${}^{3}S_{1}*$ and 3p${}^{5}$4p ${}^{3}S_{1}*$ exchanged and then the three levels are labeled as 3d${}^{9}$4d ${}^{3}D_{1}$, 3p${}^{5}$4p ${}^{3}S_{1}*$, and 3d${}^{9}$4d ${}^{3}S_{1}*$ at Z = 88. Furthermore, the unique notations 3d${}^{9}$4d ${}^{3}D_{1}$ and 3p${}^{5}$4p ${}^{3}S_{1}*$ exchange with each other at Z = 92. However, the 6th level is uniquely relabeled as 3d${}^{9}$4d ${}^{3}P_{1}$ at Z = 91 and 92, so the levels are named as the unique notations 3d${}^{9}$4d ${}^{3}D_{1}$, 3p${}^{5}$4p ${}^{3}S_{1}*$, and 3d${}^{9}$4d ${}^{3}P_{1}$ at Z = 91 and 3p${}^{5}$4p ${}^{3}S_{1}*$, 3d${}^{9}$4d ${}^{3}D_{1}$ and 3d${}^{9}$4d ${}^{3}P_{1}$ at Z = 92, respectively. In a word, the information of the 5th level exchanges with the 6th and 4th levels at Z = 88 and 92 in turn. Figure 2 gives the Shannon entropies of the 8th, 9th, 10th, and 11th levels. Their sudden changes take place at Z = 38, 70, 75, 81, and 86, where their unique notation exchanges as well. Definitely, the 10th level exchanges its unique notation with 11th at Z = 38 and 70 twice, 9th level at Z = 75, and then the 11th level at Z = 81 in turn, while the unique notations of 8th and 9th exchange with each other at Z = 86.

**Figure 3:**
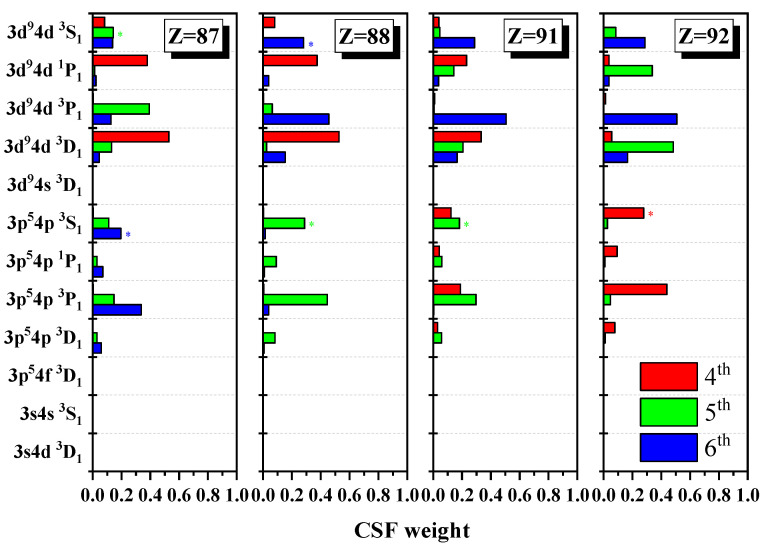
Weights of configuration state function in LS-coupled configuration space for the 4th, 5th, and 6th levels of Ni-like ions with Z = 87, 88, 91, and 92.

**Figure 4:**
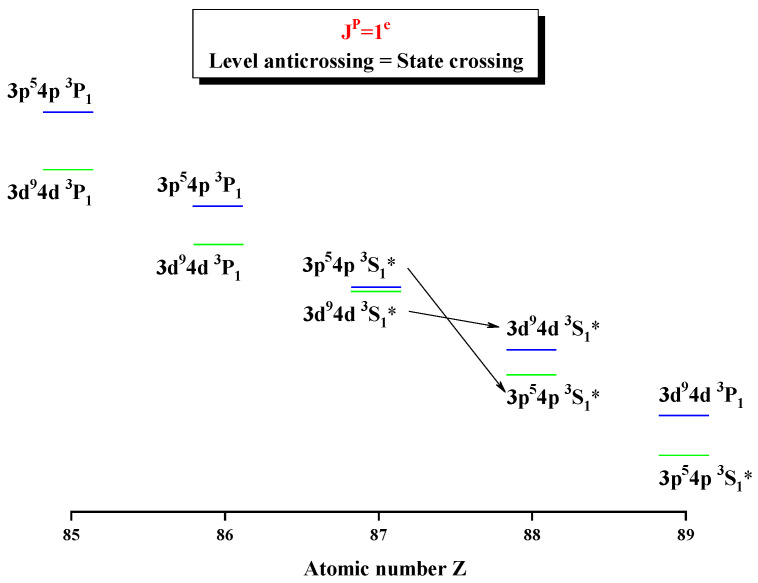
Energy diagrams for the 5th and 6th levels for Ni-like isoelectronic sequence between Z = 87 and 88.

In order to show the unique notation and information exchange for the levels at some certain Z, Table 3, Table 4, Table 5 and Table 6 present the configuration mixing coefficients, which are written in a bold font for the unique notations and Figure 3 shows the configuration weights for the atomic state functions of the 4th, 5th, and 6th levels in the subspace with $J^{P}=1^{e}$. From Table 3, it can be seen that for Z = 87, the 5th and 6th levels should be labeled as the unique notations 3d${}^{9}$4d ${}^{3}S_{1}$ and 3p${}^{5}$4p ${}^{3}S_{1}$ with “*” instead of their dominant components 3d${}^{9}$4d ${}^{3}P_{1}$ and 3p${}^{5}$4p ${}^{3}P_{1}$, respectively. In Table 4, at Z = 88, their unique notations become 3p${}^{5}$4p ${}^{3}S_{1}$ and 3d${}^{9}$4d ${}^{3}S_{1}$, which shows information exchange between the 5th and 6th levels. In Table 5, for Z = 91, the 2nd and 5th levels should be labeled as unique notations 3d${}^{9}$4d ${}^{3}S_{1}$ and 3p${}^{5}$4p ${}^{3}S_{1}$ with “*” instead of their dominant components 3d${}^{9}$4d ${}^{3}P_{1}$ and 3p${}^{5}$4p ${}^{3}P_{1}$, respectively. In Table 6, for Z = 92, the 2nd and 4th levels should be labeled as the unique notations 3d${}^{9}$4d ${}^{3}S_{1}$ and 3p${}^{5}$4p ${}^{3}S_{1}$ instead of their dominant components 3d${}^{9}$4d ${}^{3}P_{1}$ and 3p${}^{5}$4p ${}^{3}P_{1}$, respectively. Apparently, the information exchanges between the 4th and 5th levels, while there is just the unique notation for the 2nd level. In fact, as shown in Figure 3, it is necessary to use the unique notation for those levels that have much uncertain information with the remarkably nonlocalized distribution of configuration weights, and this nonlocality can measure the purity of atomic state functions. In addition, it can be noted that the sharp maxima in Shannon entropy indicate strong nonlocality in the distribution of configuration weights in a given configuration space, whether for LS or jj coupling [25]. It is shown that around Z = 87, it is not suitable to describe the 5th and 6th levels in LS and jj-coupled configuration spaces due to the strong delocalization of the distribution of configuration weights, while around Z = 91, LS and jj-coupled configuration basis sets are also not suitable for the 4th and 5th levels. Figure 4 gives the energy diagrams for the 5th and 6th levels, both of which anticross at Z = 87. Unlikely in a jj-coupled configuration space, around the anticrossing, the two levels must be relabeled uniquely. In order to avoid repetition, only the Shannon entropies and information exchanges are described in the figures below.

**Figure 5:**
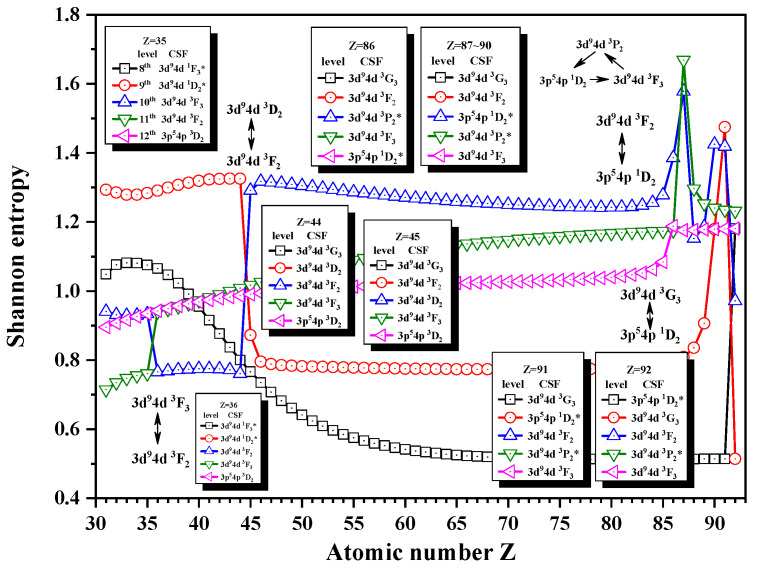
Shannon entropies for the 8th, 9th, 10th, 11th, and 12th levels in the LS-coupled subspace with $J^{P}=2^{e}$ and 3^*e*^ for a Ni-like isoelectronic sequence with Z = 31–92.

**Figure 6:**
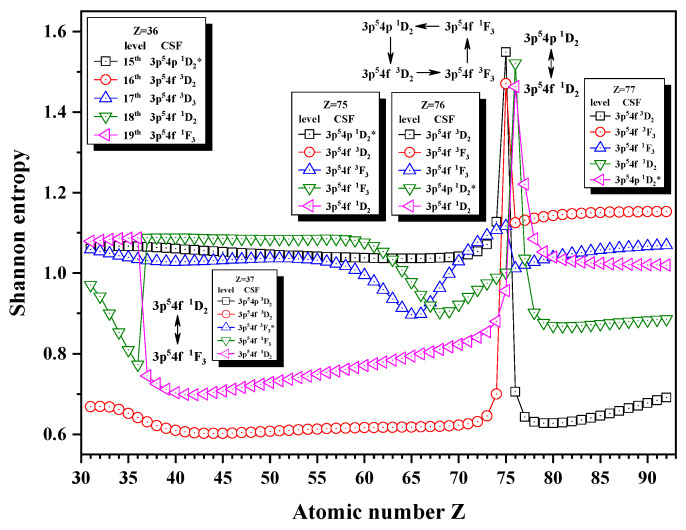
Shannon entropies for the 15th, 16th, 17th, 18th, and 19th levels in the LS-coupled subspace with $J^{P}=2^{e}$ and 3^*e*^ for a Ni-like isoelectronic sequence with Z = 31–92.

**Figure 7:**
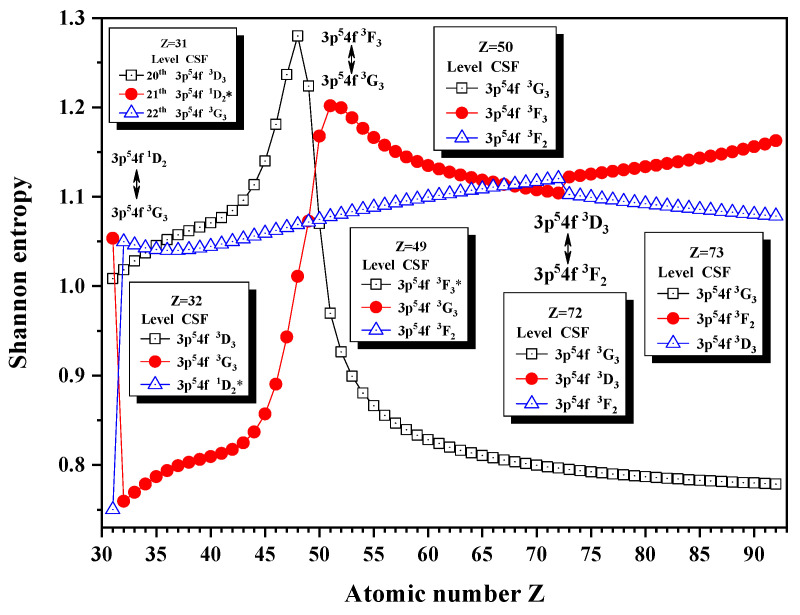
Shannon entropies for the 20th, 21th, and 22nd levels in the LS-coupled subspace with $J^{P}=2^{e}$ and 3^*e*^ for a Ni-like isoelectronic sequence with Z = 31–92.

Figure 5, Figure 6 and Figure 7 show the Shannon entropies for thirteen levels in a combined configuration space with $J^{P}=2^{e}$ and 3^*e*^. Figure 5 gives the Shannon entropies for the 8th, 9th, 10th, 11th, and 12th levels. The sudden changes take place at Z = 36, 45, 87, 91, and 92 in turn. According to their configuration mixing coefficients, the information of the 9th level exchanges with that of the 10th level at Z = 45 and 91 twice and with the 8th level at Z = 92. The information of the 11th level exchanges with that of the 10th level at Z = 36, and it can also be found that the unique notations 3d${}^{9}$4d ${}^{3}P_{2}$, 3p${}^{5}$4p ${}^{1}D_{2}$, and 3d${}^{9}$4d ${}^{3}F_{3}$ have a triangle rotation for the 10th, 11th, and 12th levels at Z = 87. Figure 6 shows the Shannon entropies for the 15th, 16th, 17th, 18th, and 19th levels. It is clear that the sudden changes take place at Z = 37, 76, and 77. The information exchanges between the 18th and 19th levels at Z = 37 and 77 twice, while at Z = 76, a quadrilateral rotation appears for the 15th, 16th, 17th, and 18th levels with the unique notations 3p${}^{5}$4f ${}^{1}D_{2}$, 3p${}^{5}$4f ${}^{3}D_{2}$, 3p${}^{5}$4f ${}^{3}F_{3}$, and 3p${}^{5}$4f ${}^{1}F_{3}$. In Figure 7, the sudden changes take place at Z = 32, 50, and 73. The information of the 21th level exchanges with that of the 22nd level at Z = 32, the 20th level at Z = 50, and the 22nd level at Z = 77 again.

**Figure 8:**
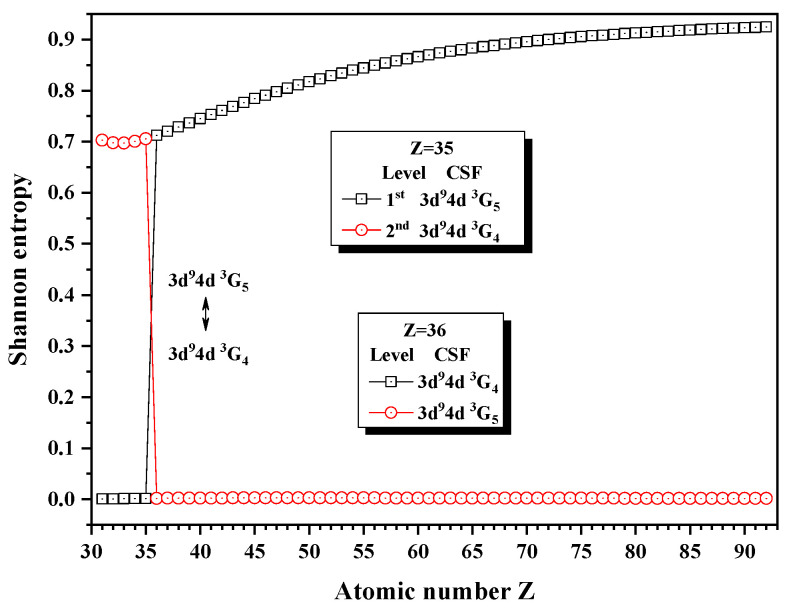
Shannon entropies for the 1st and 2nd levels in the LS-coupled subspace with $J^{P}=4^{e}$ and 5^*e*^ for a Ni-like isoelectronic sequence with Z = 31–92.

**Figure 9:**
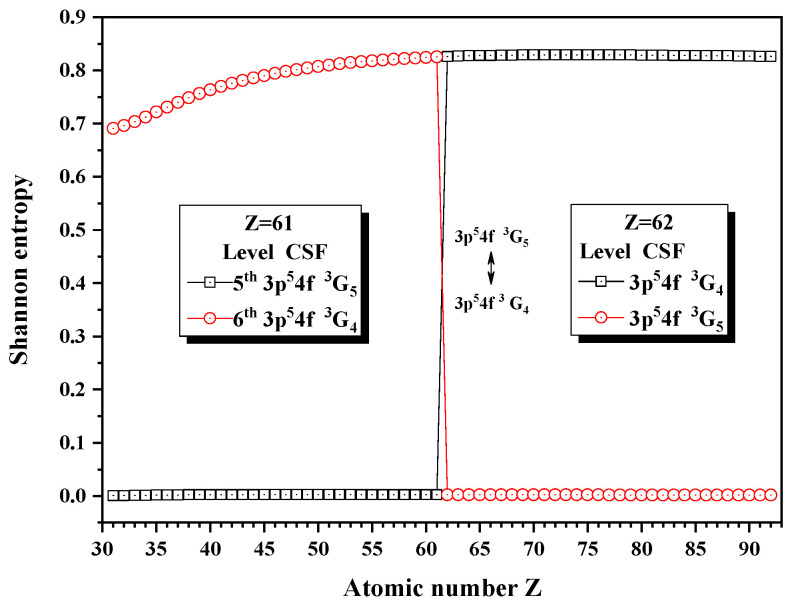
Shannon entropies for the 5th and 6th levels in the LS-coupled subspace with $J^{P}=4^{e}$ and 5^*e*^ for a Ni-like isoelectronic sequence with Z = 31–92.

**Figure 10:**
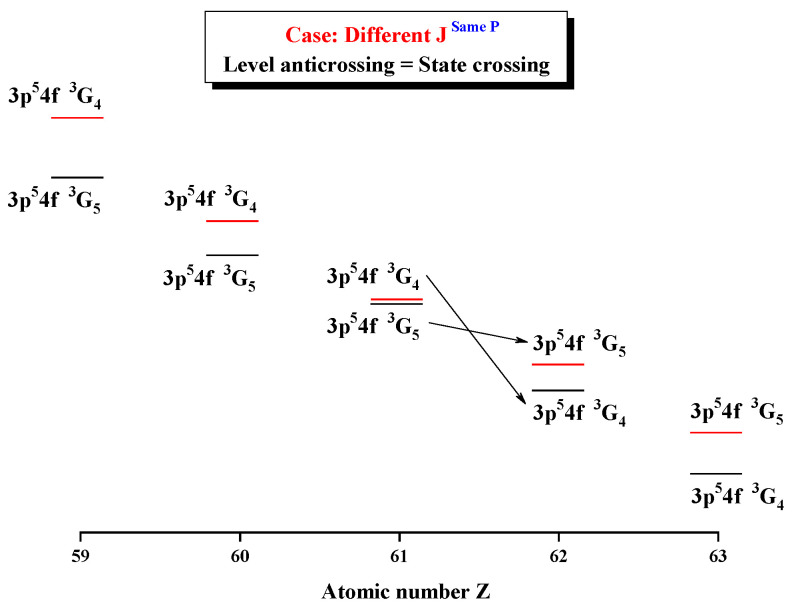
Energy diagrams for the 5th and 6th levels in the LS-coupled subspace with $J^{P}=4^{e}$ and 5^*e*^ for a Ni-like isoelectronic sequence with Z = 59–63.

Figure 8 and Figure 9 give the Shannon entropies for the 1st, 2nd, 5th, and 6th levels in the subspace with $J^{P}=4^{e}$ and 5^*e*^. In Figure 8, the entropies jump at Z = 36, where the unique notations 3d${}^{9}$4d ${}^{3}G_{5}$ and 3d${}^{9}$4d ${}^{3}G_{4}$ exchange. Figure 9 shows that the entropies jump at Z = 62, where the unique notations 3p${}^{5}$4f ${}^{3}G_{5}$ and 3p${}^{5}$4f ${}^{3}G_{4}$ exchange with each other. By the way, the 5th and 6th levels, respectively, with $J^{P}=4^{e}$ and 5^*e*^, anticross at Z = 61, as given in Figure 10. It is shown that, likely in jj-coupled configuration space [25], the levels with the different *J* and the same *P*, which do not have configuration interaction, can also anticross in the combined subspace besides those levels with the same $J^{P}$ which can interact with each other.

**Figure 11:**
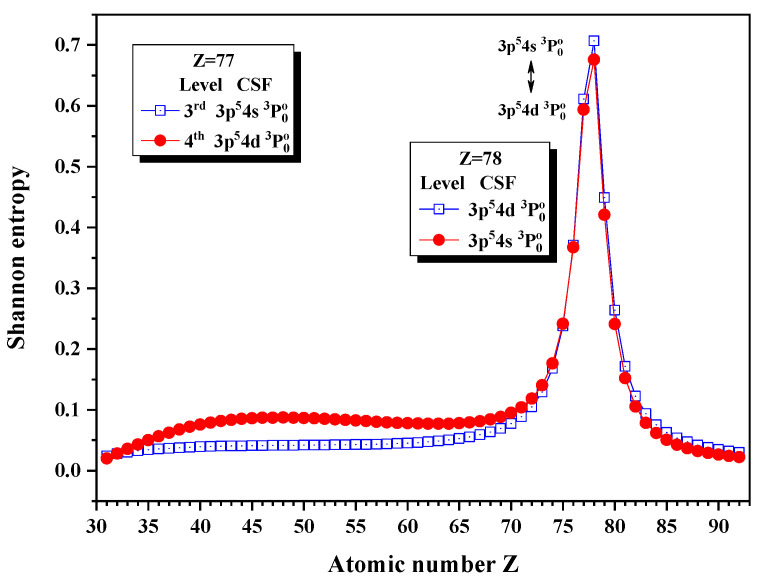
Shannon entropies for the 3rd and 4th levels in the LS-coupled subspace with $J^{P}=0^{o}$ for a Ni-like isoelectronic sequence with Z = 31–92.

Figure 11 gives the Shannon entropies for the 3rd and 4th levels in the subspace with $J^{P}=0^{o}$. Both of them have the maxima at Z = 78, where the unique notations 3p${}^{5}$4s ${}^{3}P_{0}^{o}$ and 3p${}^{5}$4d ${}^{3}P_{0}^{o}$ exchange.

**Figure 12:**
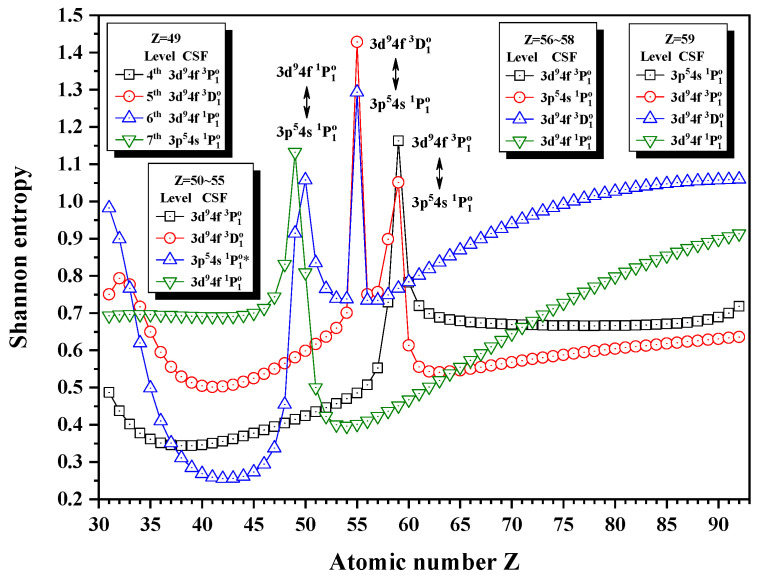
Shannon entropies for the 4th, 5th, 6th, and 7th levels in the LS-coupled subspace with $J^{P}=1^{o}$ for a Ni-like isoelectronic sequence with Z = 31–92.

**Figure 13:**
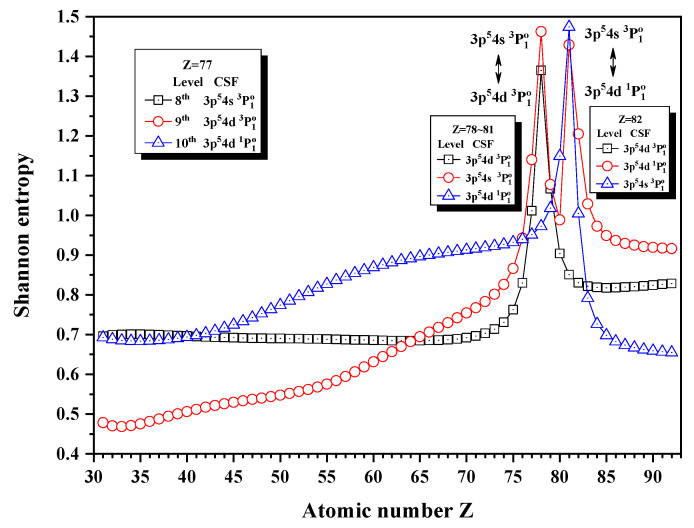
Shannon entropies for the 8th, 9th, and 10th levels in the LS-coupled subspace with $J^{P}=1^{o}$ for a Ni-like isoelectronic sequence with Z = 31–92.

**Figure 14:**
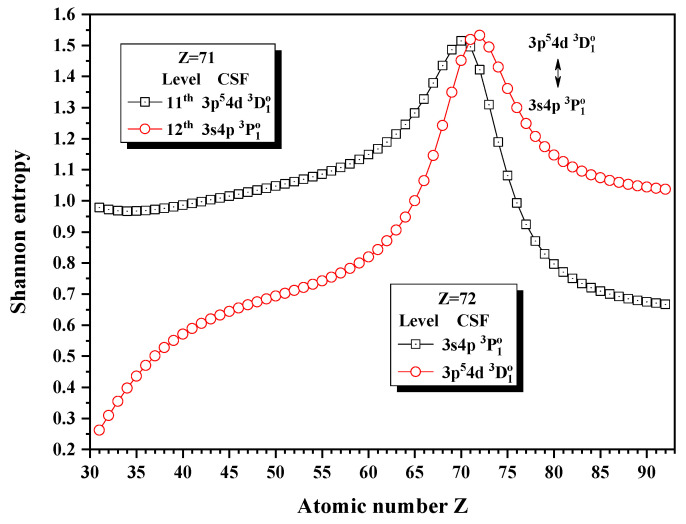
Shannon entropies for the 11th and 12th levels in the LS-coupled subspace with $J^{P}=1^{o}$ for a Ni-like isoelectronic sequence with Z = 31–92.

Figure 12, Figure 13 and Figure 14 give the Shannon entropies for the 4th, 5th, 6th, 7th, 8th, 9th, 10th, 11th, and 12th levels in the subspace with $J^{P}=1^{o}$. In Figure 12, the entropies have one maximum at Z = 49 for the 7th level, two maxima at Z = 50 and 55 for the 6th level, two maxima at Z = 55 and 59 for the 5th level, and one maximum at Z = 59 for the 4th level. Meanwhile, the unique notations 3d${}^{9}$4f ${}^{1}P_{1}^{o}$, 3d${}^{9}$4f ${}^{3}D_{1}^{o}$, and 3d${}^{9}$4f ${}^{3}P_{1}^{o}$ exchange with 3p${}^{5}$4s ${}^{1}P_{1}^{o}$ in turn at Z = 50, 56, and 59. In Figure 13, the entropies have one, two, and one maxima for the 8th, 9th, and 10th levels at Z = 78 and 81, where the unique notation 3p${}^{5}$4d ${}^{3}P_{1}^{o}$ exchanges with 3p${}^{5}$4s ${}^{3}P_{1}^{o}$ and 3p${}^{5}$4d ${}^{1}P_{1}^{o}$. In Figure 14, both the entropies of the 11th and 12th levels have maxima around Z = 70, where the unique notations 3p${}^{5}$4d ${}^{3}D_{1}^{o}$ and 3s4p ${}^{3}P_{1}^{o}$ exchange.

**Figure 15:**
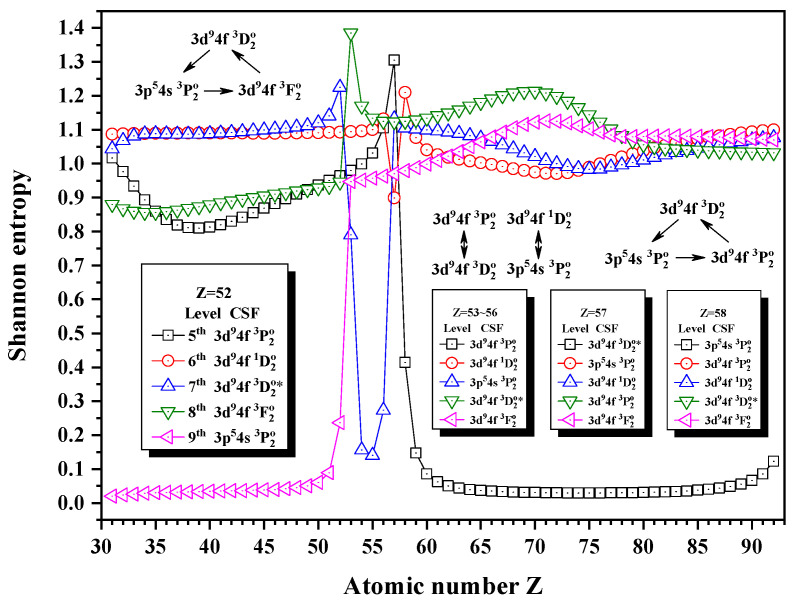
Shannon entropies for the 5th, 6th, 7th, 8th, and 9th levels in the LS-coupled subspace with $J^{P}=2^{o}$ for Ni-like isoelectronic sequence with Z = 31–92.

Figure 15 gives the Shannon entropies for the 5th, 6th, 7th, 8th, and 9th levels in the subspace with $J^{P}=2^{o}$. In Figure 15, the sudden changes of the entropies of the 7th, 8th, and 9th levels take place at Z = 53. It is interesting that the unique notations 3d${}^{9}$4f ${}^{3}D_{2}^{o}$, 3d${}^{9}$4f ${}^{3}F_{2}^{o}$, and 3p${}^{5}$4s ${}^{3}P_{2}^{o}$ form a triangle exchange at Z = 53. There are maxima at Z = 57 for the 5th level and at Z = 58 for the 6th level. Meanwhile, the unique notations 3d${}^{9}$4f ${}^{3}P_{2}^{o}$ and 3d${}^{9}$4f ${}^{3}D_{2}^{o}$, 3d${}^{9}$4f ${}^{1}D_{2}^{o}$ and 3p${}^{5}$4s ${}^{3}P_{2}^{o}$ exchange at Z = 57, while the unique notations 3d${}^{9}$4f ${}^{3}D_{2}^{o}$, 3p${}^{5}$4s ${}^{3}P_{2}^{o}$, and 3d${}^{9}$4f ${}^{3}F_{2}^{o}$ form a triangle exchange at Z = 58.

**Figure 16:**
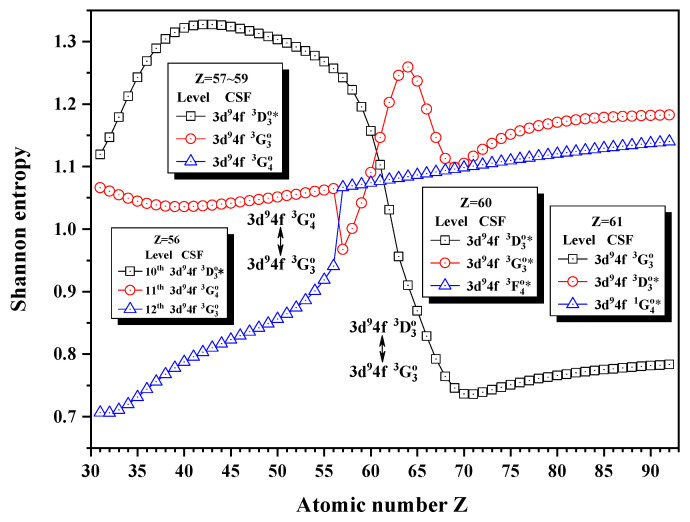
Shannon entropies for the 10th, 11th, and 12th levels in the LS-coupled subspace with $J^{P}=3^{o}$ and $4^{o}$ for a Ni-like isoelectronic sequence with Z = 31–92.

**Figure 17:**
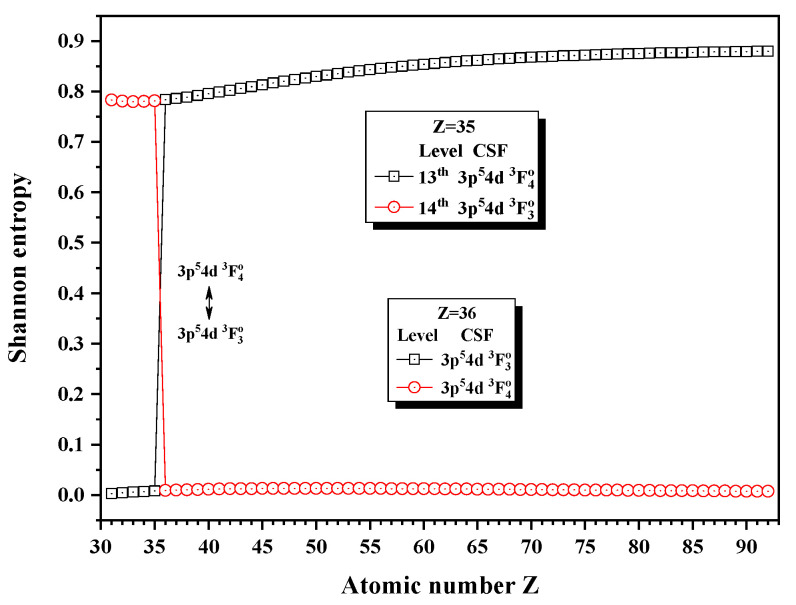
Shannon entropies for the 13th and 14th levels in the LS-coupled subspace with $J^{P}=3^{o}$ and $4^{o}$ for a Ni-like isoelectronic sequence with Z = 31–92.

**Figure 18:**
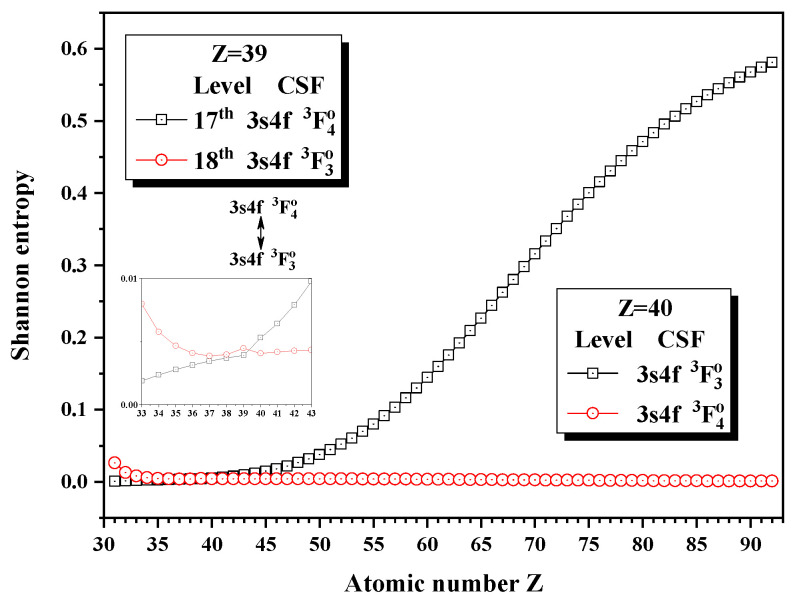
Shannon entropies for the 17th and 18th levels in the LS-coupled subspace with $J^{P}=3^{o}$ and $4^{o}$ for a Ni-like isoelectronic sequence with Z = 31–92.

Figure 16, Figure 17 and Figure 18 give the Shannon entropies for the 10th, 11th, 12th, 13th, 14th, 17th, and 18th levels in the combined subspace with $J^{P}=3^{o}$ and $4^{o}$. In Figure 16, the sudden changes of the entropies of the 11th and 12th levels take place at Z = 57, where the unique notations 3d${}^{9}$4f ${}^{3}G_{4}^{o}$ and 3d${}^{9}$4f ${}^{3}G_{3}^{o}$ exchange. Around Z = 64, the entropy has maxima for the 11th level and the unique notations 3d${}^{9}$4f ${}^{3}D_{3}^{o}$ and 3d${}^{9}$4f ${}^{3}G_{3}^{o}$ for the 10th and 11th levels exchange at Z = 60. Since the information of the 10th, 11th, and 12th levels is very uncertain due to strong configuration interaction, especially for 12th level, its unique notation is 3d${}^{9}$4f ${}^{3}G_{4}^{o}$ for Z = 57–59, at Z = 60 becomes 3d${}^{9}$4f ${}^{3}F_{4}^{o}$ and then 3d${}^{9}$4f ${}^{1}G_{4}^{o}$ at Z = 61. Figure 17 shows that the entropies of the 13th and 14th levels jump at Z = 36, where the unique notations 3p${}^{5}$4d ${}^{3}F_{4}^{o}$ and 3p${}^{5}$4d ${}^{3}F_{3}^{o}$ exchange. Similarly, Figure 18 presents the entropies of the 17th and 18th levels jump at Z = 40, where the unique notations 3s4f ${}^{3}F_{4}^{o}$ and 3s4f ${}^{3}F_{3}^{o}$ exchange.

**Figure 19:**
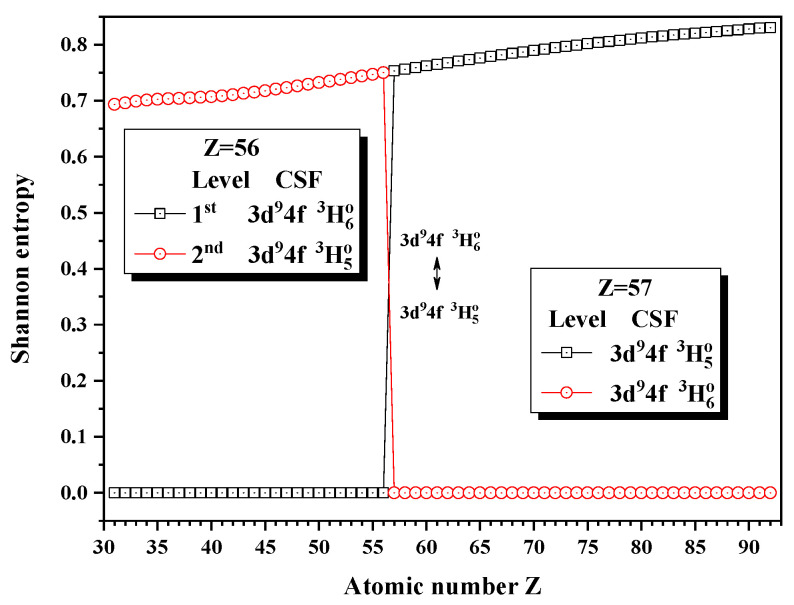
Shannon entropies for the 1st and 2nd levels labeled as 3d${}^{9}$4f ${}^{3}H_{6}^{o}$ and 3d${}^{9}$4f ${}^{3}H_{5}^{o}$ in the LS-coupled subspace with $J^{P}=5^{o}$ and $6^{o}$ for a Ni-like isoelectronic sequence with Z = 31–92.

Figure 19 presents the entropies of the 1st and 2nd levels jump at Z = 57, where the unique notations 3d${}^{9}$4f ${}^{3}H_{6}^{o}$ and 3d${}^{9}$4f ${}^{3}H_{5}^{o}$ exchange.

Table 7, Table 8, Table 9, Table 10, Table 11, Table 12, Table 13, Table 14 and Table 15 show the sudden change of Shannon entropies, information exchanges, eigenlevel anticrossings, and configuration mixing coefficients for adjacent levels, where the atomic state functions are expressed by the first three CSF components. In these tables, the sudden change is labeled as “Yes” or “No”, and the eigenlevel anticrossings are the minima of the energy difference between two adjacent levels. In Table 7, it is clear that there is no sudden change, eigenlevel anticrossing, and information exchange at Z = 48 and 49 for the 2nd and 3rd levels, which can be described by an LS-coupled configuration basis set certainly, while in Table 6 in [25], we can see there is information exchange but no sudden change and eigenlevel anticrossing at Z = 48 and 49 for the 2nd and 3rd levels, the information of which were uncertain in the jj-coupled configuration basis set. In other tables, only the information exchange related to sudden change and anticrossing is listed. Information exchanges unrelated to sudden change and anticrossing are not given. As stated in [25], from these tables where there is a sudden change in Shannon entropy, there is an eigenlevel anticrossing and vice versa. At the same time, if there is a sudden change or an eigenlevel anticrossing, there is an information exchange. In addition, it is also clarified that there is no necessary connection between a strong configuration interaction and eigenlevel anticrossing even and information exchange, because the eigenlevel anticrossing is determined by the Hamiltonian itself, which is independent of the configuration basis set in different coupling schemes.

## 4. Summary and Outlook

Based on the transformation between jj- and LS-coupled configuration basis sets, the Shannon entropies in the LS-coupled configuration space have been obtained for the ground and excited states of a Ni-like isoelectronic sequence. As we already know, the role of Shannon entropy can be considered as an information measurement of an atomic state in a given configuration space. The smaller the entropy, the more localization in the distribution of configuration weights, the more certain the information of the energy level, and the more meaningful the configuration. However, in the LS-coupled configuration space, the Shannon entropy is generally higher than that in the jj-coupled configuration space for the ground and single excited states of most of Ni-like ions. Large Shannon entropy stands for the delocalization of configuration weights, which describes the extent of configuration interaction in a certain atomic state function. If there is a strong configuration interaction, it may be invalid that the energy level is labeled by the dominant component in a given configuration space. As a result of this, the unique algorithm has to be used to label the level uniquely in order to describe their information. On the basis of unique notation, a relationship has also been found among the sudden change of Shannon entropy, information exchange, eigenlevel anticrossing, and strong configuration interaction in LS-coupled configuration space.

As we hope, the conclusion is exactly the same as that of our previous work [25], but the situation is more complicated than before, especially for information exchange. Firstly, the sudden change of Shannon entropy in an LS-coupled configuration space is a sufficient and necessary condition for the eigenlevel anticrossing, which means that the sudden change of Shannon entropy can be considered as the effective indicator of the eigenlevel anticrossing likely in a jj-coupled configuration space. Secondly, if there are sudden changes of Shannon entropy in an LS-coupled configuration space and eigenlevel anticrossings, information exchange must take place, which is very much the same in a jj-coupled configuration space. Thirdly, Shannon entropy describes the nonlocality of configuration expansion in an atomic state function, which can reflect the strength of configuration interaction. Compared with the Shannon entropy in an LS-coupled configuration space, the Shannon entropy is so small in a jj-coupled configuration space that the information of most energy levels is certain and can be labeled by their dominant component. On the contrary, the Shannon entropy in an LS-coupled configuration space is larger for the levels of most of Ni-like ions, so the unique notation has to be used in order to describe the information of energy levels. In addition, there is much more information exchange in an LS-coupled configuration space than that in a jj-coupled configuration space but no sudden change of Shannon entropy and eigenlevel anticrossing. In a word, the structure of Shannon entropy along the Ni-like isoelectronic sequence can not be changed due to the LS-jj transformation; the structure of sudden change is invariant. Furthermore, it is expected that in a proper coupling scheme (e.g., one of LS, jj, LK, and jK coupling schemes or others) the structure of sudden change of Shannon entropy would be shown remarkably.

## Figures and Tables

**Table 1:** LS-coupled subspaces expanded by the ground and single excited configuration state functions with $J^{P}=0^{e},1^{e},2^{e},3^{e},4^{e}$, and 5^*e*^.

0^*e*^	1^*e*^	2^*e*^	3^*e*^	4^*e*^	5^*e*^
3d${}^{10}$ ${}^{1}S_{0}$	3d${}^{9}$4d ${}^{3}S_{1}$	3d${}^{9}$4s ${}^{1}D_{2}$	3d${}^{9}$4s ${}^{3}D_{3}$	3d${}^{9}$4d ${}^{3}F_{4}$	3d${}^{9}$4d ${}^{3}G_{5}$
3d${}^{9}$4d ${}^{1}S_{0}$	3d${}^{9}$4d ${}^{1}P_{1}$	3d${}^{9}$4s ${}^{3}D_{2}$	3d${}^{9}$4d ${}^{3}D_{3}$	3d${}^{9}$4d ${}^{1}G_{4}$	3p${}^{5}$4f ${}^{3}G_{5}$
3d${}^{9}$4d ${}^{3}P_{0}$	3d${}^{9}$4d ${}^{3}P_{1}$	3d${}^{9}$4d ${}^{3}P_{2}$	3d${}^{9}$4d ${}^{1}F_{3}$	3d${}^{9}$4d ${}^{3}G_{4}$	
3p${}^{5}$4p ${}^{1}S_{0}$	3d${}^{9}$4d ${}^{3}D_{1}$	3d${}^{9}$4d ${}^{1}D_{2}$	3d${}^{9}$4d ${}^{3}F_{3}$	3p${}^{5}$4f ${}^{3}F_{4}$	
3p${}^{5}$4p ${}^{3}P_{0}$	3d${}^{9}$4s ${}^{3}D_{1}$	3d${}^{9}$4d ${}^{3}D_{2}$	3d${}^{9}$4d ${}^{3}G_{3}$	3p${}^{5}$4f ${}^{1}G_{4}$	
3s4s ${}^{1}S_{0}$	3p${}^{5}$4p ${}^{3}S_{1}$	3d${}^{9}$4d ${}^{3}F_{2}$	3p${}^{5}$4p ${}^{3}D_{3}$	3p${}^{5}$4f ${}^{3}G_{4}$	
	3p${}^{5}$4p ${}^{1}P_{1}$	3p${}^{5}$4p ${}^{3}P_{2}$	3p${}^{5}$4f ${}^{3}D_{3}$		
	3p${}^{5}$4p ${}^{3}P_{1}$	3p${}^{5}$4p ${}^{1}D_{2}$	3p${}^{5}$4f ${}^{1}F_{3}$		
	3p${}^{5}$4p ${}^{3}D_{1}$	3p${}^{5}$4p ${}^{3}D_{2}$	3p${}^{5}$4f ${}^{3}F_{3}$		
	3p${}^{5}$4f ${}^{3}D_{1}$	3p${}^{5}$4f ${}^{1}D_{2}$	3p${}^{5}$4f ${}^{3}G_{3}$		
	3s4s ${}^{3}S_{1}$	3p${}^{5}$4f ${}^{3}D_{2}$	3s4d ${}^{3}D_{3}$		
	3s4d ${}^{3}D_{1}$	3p${}^{5}$4f ${}^{3}F_{2}$			
		3s4d ${}^{1}D_{2}$			
		3s4d ${}^{3}D_{2}$			
$n_{c}\left(0^{e}\right)=6$	$n_{c}\left(1^{e}\right)=12$	$n_{c}\left(2^{e}\right)=14$	$n_{c}\left(3^{e}\right)=11$	$n_{c}\left(4^{e}\right)=6$	$n_{c}\left(5^{e}\right)=2$

**Table 2:** LS-coupled subspaces expanded by the single excited configuration state functions with $J^{P}=0^{o},1^{o},2^{o},3^{o},4^{o},5^{o}$, and $6^{o}$.

0${}^{o}$	1${}^{o}$	2${}^{o}$	3${}^{o}$	4${}^{o}$	5${}^{o}$	6${}^{o}$
3d${}^{9}$4f ${}^{3}P_{0}^{o}$	3d${}^{9}$4p ${}^{1}P_{1}^{o}$	3d${}^{9}$4p ${}^{3}P_{2}^{o}$	3d${}^{9}$4p ${}^{3}D_{3}^{o}$	3d${}^{9}$4p ${}^{3}F_{4}^{o}$	3d${}^{9}$4f ${}^{3}G_{5}^{o}$	3d${}^{9}$4f ${}^{3}H_{6}^{o}$
3d${}^{9}$4p ${}^{3}P_{0}^{o}$	3d${}^{9}$4p ${}^{3}P_{1}^{o}$	3d${}^{9}$4p ${}^{1}D_{2}^{o}$	3d${}^{9}$4p ${}^{1}F_{3}^{o}$	3d${}^{9}$4f ${}^{3}F_{4}^{o}$	3d${}^{9}$4f ${}^{1}H_{5}^{o}$	
3p${}^{5}$4d ${}^{3}P_{0}^{o}$	3d${}^{9}$4p ${}^{3}D_{1}^{o}$	3d${}^{9}$4p ${}^{3}D_{2}^{o}$	3d${}^{9}$4p ${}^{3}F_{3}^{o}$	3d${}^{9}$4f ${}^{1}G_{4}^{o}$	3d${}^{9}$4f ${}^{3}H_{5}^{o}$	
3p${}^{5}$4s ${}^{3}P_{0}^{o}$	3d${}^{9}$4f ${}^{1}P_{1}^{o}$	3d${}^{9}$4p ${}^{3}F_{2}^{o}$	3d${}^{9}$4f ${}^{3}D_{3}^{o}$	3d${}^{9}$4f ${}^{3}G_{4}^{o}$		
3s4p ${}^{3}P_{0}^{o}$	3d${}^{9}$4f ${}^{3}P_{1}^{o}$	3d${}^{9}$4f ${}^{3}P_{2}^{o}$	3d${}^{9}$4f ${}^{1}F_{3}^{o}$	3d${}^{9}$4f ${}^{3}H_{4}^{o}$		
	3d${}^{9}$4f ${}^{3}D_{1}^{o}$	3d${}^{9}$4f ${}^{1}D_{2}^{o}$	3d${}^{9}$4f ${}^{3}F_{3}^{o}$	3p${}^{5}$4d ${}^{3}F_{4}^{o}$		
	3p${}^{5}$4s ${}^{1}P_{1}^{o}$	3d${}^{9}$4f ${}^{3}D_{2}^{o}$	3d${}^{9}$4f ${}^{3}G_{3}^{o}$	3s4f ${}^{3}F_{4}^{o}$		
	3p${}^{5}$4s ${}^{3}P_{1}^{o}$	3d${}^{9}$4f ${}^{3}F_{2}^{o}$	3p${}^{5}$4d ${}^{3}D_{3}^{o}$			
	3p${}^{5}$4d ${}^{1}P_{1}^{o}$	3p${}^{5}$4s ${}^{3}P_{2}^{o}$	3p${}^{5}$4d ${}^{1}F_{3}^{o}$			
	3p${}^{5}$4d ${}^{3}P_{1}^{o}$	3p${}^{5}$4d ${}^{3}P_{2}^{o}$	3p${}^{5}$4d ${}^{3}F_{3}^{o}$			
	3p${}^{5}$4d ${}^{3}D_{1}^{o}$	3p${}^{5}$4d ${}^{1}D_{2}^{o}$	3s4f ${}^{1}F_{3}^{o}$			
	3s4p ${}^{1}P_{1}^{o}$	3p${}^{5}$4d ${}^{3}D_{2}^{o}$	3s4f ${}^{3}F_{3}^{o}$			
	3s4p ${}^{3}P_{1}^{o}$	3p${}^{5}$4d ${}^{3}F_{2}^{o}$				
		3s4p ${}^{3}P_{2}^{o}$				
		3s4f ${}^{3}F_{2}^{o}$				
$n_{c}\left(0^{o}\right)=5$	$n_{c}\left(1^{o}\right)=13$	$n_{c}\left(2^{o}\right)=15$	$n_{c}\left(3^{o}\right)=12$	$n_{c}\left(4^{o}\right)=7$	$n_{c}\left(5^{o}\right)=3$	$n_{c}\left(6^{o}\right)=1$

**Table 3:** Configuration mixing coefficients for the 12 levels in the LS-coupled subspace with $J^{P}=1^{e}$ at Z = 87.

CSF	1st	2nd	3rd	4th	5th	6th	7th	8th	9th	10th	11th	12th
3d${}^{9}$4d ${}^{3}S_{1}$	−0.0002	−0.6231	−0.4958	−0.2877	**−0.3779** *	0.3717	−0.0431	−0.0013	−0.0073	0.0139	0.0114	0.0000
3d${}^{9}$4d ${}^{1}P_{1}$	0.0010	0.1521	**−0.7492**	0.6148	0.1188	−0.1502	−0.0255	0.0024	0.0102	−0.0014	−0.0049	0.0000
3d${}^{9}$4d ${}^{3}P_{1}$	0.0014	**0.6852**	−0.0803	−0.0591	−0.6264	0.3578	−0.0076	0.0003	−0.0001	0.0036	0.0107	0.0001
3d${}^{9}$4d ${}^{3}D_{1}$	−0.0121	−0.3303	0.4292	**0.7272**	−0.3626	0.2141	0.0075	0.0097	0.0152	0.0014	0.0041	0.0039
3d${}^{9}$4s ${}^{3}D_{1}$	**0.9998**	−0.0060	0.0061	0.0078	−0.0081	−0.0041	−0.0050	−0.0029	−0.0104	−0.0010	−0.0030	−0.0023
3p${}^{5}$4p ${}^{3}S_{1}$	−0.0010	−0.0661	−0.0346	−0.0525	−0.3332	**−0.4436** *	0.6005	0.0319	0.1844	−0.0861	−0.5299	0.0010
3p${}^{5}$4p ${}^{1}P_{1}$	0.0022	0.0214	-0.0273	0.0349	0.1726	0.2660	**0.7453**	−0.0872	−0.4700	0.0733	0.3282	0.0023
3p${}^{5}$4p ${}^{3}P_{1}$	0.0034	0.0664	0.0053	0.0524	0.3857	0.5809	0.0208	−0.0039	0.0017	−0.1666	**−0.6916**	0.0038
3p${}^{5}$4p ${}^{3}D_{1}$	−0.0141	−0.0257	0.0109	−0.0071	−0.1712	−0.2456	−0.2833	−0.1534	**−0.8434**	−0.0920	−0.2922	0.0212
3p${}^{5}$4f ${}^{3}D_{1}$	0.0011	0.0021	−0.0025	−0.0050	0.0038	0.0006	0.0022	**0.9835**	−0.1799	−0.0015	−0.0032	−0.0182
3s4s ${}^{3}S_{1}$	0.0000	−0.0085	−0.0066	−0.0051	−0.0157	−0.0098	0.0256	0.0043	0.0279	**−0.9751**	0.2175	0.0000
3s4d ${}^{3}D_{1}$	0.0026	0.0016	−0.0018	−0.0030	0.0036	0.0020	0.0036	0.0213	0.0154	0.0024	0.0085	**0.9996**

**Table 4:** Configuration mixing coefficients for the 12 levels in the LS-coupled subspace with $J^{P}=1^{e}$ at Z = 88.

CSF	1st	2nd	3rd	4th	5th	6th	7th	8th	9th	10th	11th	12th
3d${}^{9}$4d ${}^{3}S_{1}$	−0.0002	−0.6207	−0.4972	−0.2855	−0.0432	**−0.5311** *	−0.0429	−0.0007	−0.0073	0.0132	0.0115	0.0000
3d${}^{9}$4d ${}^{1}P_{1}$	0.0010	0.1540	**−0.7492**	0.6130	−0.0190	0.1952	−0.0252	0.0016	0.0101	−0.0012	−0.0048	0.0001
3d${}^{9}$4d ${}^{3}P_{1}$	0.0014	**0.6858**	−0.0773	−0.0597	−0.2516	−0.6757	−0.0078	0.0003	−0.0001	0.0032	0.0105	0.0001
3d${}^{9}$4d ${}^{3}D_{1}$	−0.0119	−0.3318	0.4282	**0.7267**	−0.1563	−0.3918	0.0073	0.0084	0.0155	0.0013	0.0040	0.0038
3d${}^{9}$4s ${}^{3}D_{1}$	**0.9998**	−0.0060	0.0060	0.0075	−0.0090	−0.0019	−0.0049	−0.0021	−0.0103	−0.0009	−0.0029	−0.0022
3p${}^{5}$4p ${}^{3}S_{1}$	−0.0010	−0.0675	−0.0349	−0.0652	**−0.5379** *	0.1260	0.6009	0.0183	0.1865	−0.0713	−0.5324	0.0011
3p${}^{5}$4p ${}^{1}P_{1}$	0.0022	0.0226	−0.0267	0.0421	0.3027	−0.0936	**0.7452**	−0.0521	−0.4749	0.0632	0.3302	0.0023
3p${}^{5}$4p ${}^{3}P_{1}$	0.0034	0.0685	0.0061	0.0688	0.6673	−0.1974	0.0198	−0.0037	0.0014	−0.1457	**−0.6961**	0.0038
3p${}^{5}$4p ${}^{3}D_{1}$	−0.0138	−0.0266	0.0104	−0.0144	−0.2887	0.0788	−0.2828	−0.0903	**−0.8526**	−0.0833	−0.2948	0.0209
3p${}^{5}$4f ${}^{3}D_{1}$	0.0011	0.0021	−0.0025	−0.0049	0.0034	0.0020	0.0022	**0.9941**	−0.1064	−0.0013	−0.0029	−0.0175
3s4s ${}^{3}S_{1}$	0.0000	−0.0083	−0.0065	−0.0054	−0.0176	−0.0027	0.0248	0.0023	0.0281	**−0.9811**	0.1886	0.0000
3s4d ${}^{3}D_{1}$	0.0026	0.0016	−0.0018	−0.0029	0.0040	0.0007	0.0035	0.0194	0.0168	0.0022	0.0086	**0.9996**

**Table 5:** Configuration mixing coefficients for the 12 levels in the LS-coupled subspace with $J^{P}=1^{e}$ at Z = 91.

CSF	1st	2nd	3rd	4th	5th	6th	7th	8th	9th	10th	11th	12th
3d${}^{9}$4d ${}^{3}S_{1}$	−0.0003	**−0.6139** *	0.5007	−0.1954	0.2150	−0.5347	−0.0424	0.0003	0.0068	0.0118	−0.0111	0.0000
3d${}^{9}$4d ${}^{1}P_{1}$	0.0010	0.1590	**0.7491**	0.4815	−0.3798	0.1919	−0.0242	−0.0010	−0.0095	−0.0008	0.0044	0.0001
3d${}^{9}$4d ${}^{3}P_{1}$	0.0014	0.6872	0.0695	−0.1060	−0.0688	**−0.7119**	−0.0085	−0.0003	0.0000	0.0024	−0.0098	0.0001
3d${}^{9}$4d ${}^{3}D_{1}$	−0.0113	−0.3357	−0.4256	**0.5764**	−0.4553	−0.4075	0.0067	−0.0073	−0.0149	0.0010	−0.0037	0.0036
3d${}^{9}$4s ${}^{3}D_{1}$	**0.9998**	−0.0059	−0.0056	0.0014	−0.0108	−0.0034	−0.0047	0.0015	0.0098	−0.0006	0.0027	−0.0021
3p${}^{5}$4p ${}^{3}S_{1}$	−0.0011	−0.0727	0.0362	−0.3523	**−0.4266** *	0.0124	0.6019	−0.0074	−0.1878	−0.0425	0.5364	0.0012
3p${}^{5}$4p ${}^{1}P_{1}$	0.0023	0.0268	0.0248	0.2069	0.2438	−0.0302	**0.7452**	0.0233	0.4763	0.0426	−0.3331	0.0022
3p${}^{5}$4p ${}^{3}P_{1}$	0.0036	0.0766	−0.0090	0.4345	0.5449	−0.0544	0.0172	0.0032	−0.0010	−0.1036	**0.7031**	0.0039
3p${}^{5}$4p ${}^{3}D_{1}$	−0.0131	−0.0302	−0.0088	−0.1751	−0.2426	0.0185	−0.2814	0.0382	**0.8568**	−0.0656	0.2991	0.0199
3p${}^{5}$4f ${}^{3}D_{1}$	0.0011	0.0021	0.0024	−0.0026	0.0050	0.0026	0.0021	**−0.9988**	0.0457	−0.0008	0.0022	−0.0156
3s4s ${}^{3}S_{1}$	0.0000	−0.0078	0.0062	−0.0122	−0.0098	−0.0061	0.0226	−0.0007	−0.0281	**−0.9905**	−0.1309	0.0000
3s4d ${}^{3}D_{1}$	0.0024	0.0015	0.0016	−0.0004	0.0044	0.0014	0.0032	−0.0164	−0.0171	0.0016	−0.0085	**0.9997**

**Table 6:** Configuration mixing coefficients for the 12 levels in the LS-coupled subspace with $J^{P}=1^{e}$ at Z = 92.

CSF	1st	2nd	3rd	4th	5th	6th	7th	8th	9th	10th	11th	12th
3d${}^{9}$4d ${}^{3}S_{1}$	−0.0003	**−0.6118** *	0.5016	−0.0353	0.2904	−0.5352	−0.0422	0.0002	0.0067	0.0115	−0.0109	0.0000
3d${}^{9}$4d ${}^{1}P_{1}$	0.0010	0.1604	**0.7491**	0.1917	−0.5822	0.1918	−0.0239	−0.0009	−0.0093	−0.0007	0.0043	0.0001
3d${}^{9}$4d ${}^{3}P_{1}$	0.0014	0.6875	0.0672	−0.1202	0.0018	**−0.7129**	−0.0087	−0.0003	0.0000	0.0022	−0.0095	0.0001
3d${}^{9}$4d ${}^{3}D_{1}$	−0.0111	−0.3368	−0.4249	0.2378	**−0.6954**	−0.4068	0.0065	−0.0071	−0.0146	0.0009	−0.0036	0.0036
3d${}^{9}$4s ${}^{3}D_{1}$	**0.9998**	−0.0059	−0.0055	−0.0047	−0.0096	−0.0034	−0.0046	0.0014	0.0096	−0.0006	0.0027	−0.0021
3p${}^{5}$4p ${}^{3}S_{1}$	−0.0012	−0.0750	0.0369	**−0.5278** *	−0.1630	0.0051	0.6022	−0.0060	−0.1881	−0.0361	0.5372	0.0012
3p${}^{5}$4p ${}^{1}P_{1}$	0.0023	0.0285	0.0240	0.3075	0.0906	−0.0264	**0.7451**	0.0197	0.4763	0.0378	−0.3336	0.0022
3p${}^{5}$4p ${}^{3}P_{1}$	0.0036	0.0800	−0.0104	0.6628	0.2168	−0.0449	0.0164	0.0030	−0.0010	−0.0938	**0.7043**	0.0039
3p${}^{5}$4p ${}^{3}D_{1}$	−0.0128	−0.0317	−0.0081	−0.2799	−0.1063	0.0148	−0.2809	0.0316	**0.8572**	−0.0616	0.2999	0.0196
3p${}^{5}$4f ${}^{3}D_{1}$	0.0011	0.0021	0.0024	0.0005	0.0056	0.0026	0.0021	**−0.9991**	0.0380	−0.0007	0.0020	−0.0150
3s4s ${}^{3}S_{1}$	0.0000	−0.0077	0.0061	−0.0150	−0.0013	−0.0062	0.0219	−0.0006	−0.0281	**−0.9922**	−0.1177	0.0000
3s4d ${}^{3}D_{1}$	0.0024	0.0015	0.0016	0.0020	0.0038	0.0014	0.0031	−0.0156	−0.0170	0.0015	−0.0085	**0.9997**

**Table 7:** Types of sudden change of Shannon entropies, eigenlevel anticrossings (in a.u.), configuration mixing coefficients, and information exchanges for the levels in the LS-coupled subspace with $J^{P}=0^{e}$.

Z	Sudden Change	Eigenlevel Anticrossing	Configuration Mixing Coefficients
48	No	No	$|2\rangle :0.9943\left(3d^{9}4d{\;}^{3}P_{0}\right)$, $|3\rangle :-0.9887\left(3d^{9}4d{\;}^{1}S_{0}\right)$
49	No	No	$|2\rangle :0.9937\left(3d^{9}4d{\;}^{3}P_{0}\right)$, $|3\rangle :-0.9879\left(3d^{9}4d{\;}^{1}S_{0}\right)$

**Table 8:** Sudden change of Shannon entropies, eigenlevel anticrossings (in a.u.), configuration mixing coefficients, and information exchanges for the levels in the LS-coupled subspace with $J^{P}=1^{e}$.

Z	Sudden Change	Eigenlevel Anticrossing	Configuration Mixing Coefficients
37	No	$\Delta E_{11,10}=0.00615$	$|10\rangle :-0.9987\left(3p^{5}4f{\;}^{3}D_{1}\right)$, $|11\rangle :-0.9945\left(3s4s{\;}^{3}S_{1}\right)$
38	Yes	No	$|10\rangle :-0.9942\left(3s4s{\;}^{3}S_{1}\right)$, $|11\rangle :0.9985\left(3p^{5}4f{\;}^{3}D_{1}\right)$
69	No	No	$|10\rangle :-0.9827\left(3s4s{\;}^{3}S_{1}\right)$, $|11\rangle :0.9989\left(3p^{5}4f{\;}^{3}D_{1}\right)$
70	Yes	$\Delta E_{11,10}=0.0792$	$|10\rangle :0.9985\left(3p^{5}4f{\;}^{3}D_{1}\right)$, $|11\rangle :-0.9800\left(3s4s{\;}^{3}S_{1}\right)$
74	No	No	$|9\rangle :-0.6927\left(3p^{5}4p{\;}^{3}P_{1}\right)-0.4972\left(3p^{5}4p{\;}^{3}S_{1}*\right)+0.3283\left(3p^{5}4p{\;}^{1}P_{1}\right)$, $|10\rangle :-0.9971\left(3p^{5}4f{\;}^{3}D_{1}\right)$
75	Yes	$\Delta E_{10,9}=0.0613$	$|9\rangle :0.7579\left(3p^{5}4f{\;}^{3}D_{1}\right)-0.4496\left(3p^{5}4p{\;}^{3}P_{1}\right)-0.3254\left(3p^{5}4p{\;}^{3}S_{1}\right)$, $|10\rangle :0.6509\left(3p^{5}4f{\;}^{3}D_{1}\right)+0.5194\left(3p^{5}4p{\;}^{3}P_{1}\right)+0.3694\left(3p^{5}4p{\;}^{3}S_{1}*\right)$
80	Yes	$\Delta E_{11,10}=0.9544$	$|10\rangle :-0.6581\left(3s4s{\;}^{3}S_{1}\right)-0.5462\left(3p^{5}4p{\;}^{3}P_{1}\right)-0.3758\left(3p^{5}4p{\;}^{3}S_{1}*\right)$, $|11\rangle :0.7513\left(3s4s{\;}^{3}S_{1}\right)-0.4584\left(3p^{5}4p{\;}^{3}P_{1}\right)-0.3794\left(3p^{5}4p{\;}^{3}S_{1}\right)$
81	No	No	$|10\rangle :-0.7587\left(3s4s{\;}^{3}S_{1}\right)-0.4745\left(3p^{5}4p{\;}^{3}P_{1}\right)-0.3188\left(3p^{5}4p{\;}^{3}S_{1}\right)$, $|11\rangle :0.6495\left(3s4s{\;}^{3}S_{1}\right)-0.5319\left(3p^{5}4p{\;}^{3}P_{1}\right)-0.4290\left(3p^{5}4p{\;}^{3}S_{1}*\right)$
85	No	No	$|8\rangle :0.8019\left(3p^{5}4p{\;}^{3}D_{1}\right)+0.4483\left(3p^{5}4p{\;}^{1}P_{1}\right)-0.3518\left(3p^{5}4f{\;}^{3}D_{1}\right)$, $|9\rangle :0.9358\left(3p^{5}4f{\;}^{3}D_{1}\right)$
86	Yes	$\Delta E_{9,8}=0.3714$	$|8\rangle :0.8901\left(3p^{5}4f{\;}^{3}D_{1}\right)-0.3896\left(3p^{5}4p{\;}^{3}D_{1}\right)-0.2188\left(3p^{5}4p{\;}^{1}P_{1}\right)$, $|9\rangle :0.7633\left(3p^{5}4p{\;}^{3}D_{1}\right)+0.4552\left(3p^{5}4f{\;}^{3}D_{1}\right)+0.4253\left(3p^{5}4p{\;}^{1}P_{1}\right)$
87	Yes	$\Delta E_{6,5}=0.2609$	$|5\rangle :-0.6264\left(3d^{9}4d{\;}^{3}P_{1}\right)+0.3856\left(3p^{5}4p{\;}^{3}P_{1}\right)-0.3779\left(3d^{9}4d{\;}^{3}S_{1}*\right)$, $|6\rangle :0.5809\left(3p^{5}4p{\;}^{3}P_{1}\right)-0.4435\left(3p^{5}4p{\;}^{3}S_{1}*\right)+0.3717\left(3d^{9}4d{\;}^{3}S_{1}\right)$
88	No	No	$|5\rangle :0.6672\left(3p^{5}4p{\;}^{3}P_{1}\right)-0.5379\left(3p^{5}4p{\;}^{3}S_{1}*\right)+0.3027\left(3p^{5}4p{\;}^{1}P_{1}\right)$, $|6\rangle :-0.6757\left(3d^{9}4d{\;}^{3}P_{1}\right)-0.5310\left(3d^{9}4d{\;}^{3}S_{1}*\right)-0.3918\left(3d^{9}4d{\;}^{3}D_{1}\right)$
91	Yes	$\Delta E_{5,4}=0.3299$	$|4\rangle :0.5764\left(3d^{9}4d{\;}^{3}D_{1}\right)+0.4815\left(3d^{9}4d{\;}^{1}P_{1}\right)+0.4345\left(3p^{5}4p{\;}^{3}P_{1}\right)$, $|5\rangle :0.5449\left(3p^{5}4p{\;}^{3}P_{1}\right)-0.4553\left(3d^{9}4d{\;}^{3}D_{1}\right)-0.4266\left(3p^{5}4p{\;}^{3}S_{1}*\right)$
92	No	No	$|4\rangle :0.6627\left(3p^{5}4p{\;}^{3}P_{1}\right)-0.5278\left(3p^{5}4p{\;}^{3}S_{1}*\right)+0.3074\left(3p^{5}4p{\;}^{1}P_{1}\right)$, $|5\rangle :-0.6953\left(3d^{9}4d{\;}^{3}D_{1}\right)-0.5821\left(3d^{9}4d{\;}^{1}P_{1}\right)+0.2904\left(3d^{9}4d{\;}^{3}S_{1}\right)$

**Table 9:** Sudden change of Shannon entropies, eigenlevel anticrossings (in a.u.), configuration mixing coefficients, and information exchanges for the levels in the LS-coupled subspace with $J^{P}=2^{e}$ and 3^*e*^.

Z	Sudden Change	Eigenlevel Anticrossing	Configuration Mixing Coefficients
31	No	$\Delta E_{22,21}=3.0\times 10^{-5}$	$|21\rangle :-0.6796\left(3p^{5}4f{\;}^{3}F_{2}\right)-0.5693\left(3p^{5}4f{\;}^{1}D_{2}*\right)-0.4624\left(3p^{5}4f{\;}^{3}D_{2}\right)$, $|22\rangle :-0.8603\left(3p^{5}4f{\;}^{3}G_{3}\right)-0.3915\left(3p^{5}4f{\;}^{1}F_{3}\right)-0.3265\left(3p^{5}4f{\;}^{3}F_{3}\right)$
32	Yes	No	$|21\rangle :-0.8571\left(3p^{5}4f{\;}^{3}G_{3}\right)-0.3965\left(3p^{5}4f{\;}^{1}F_{3}\right)-0.3286\left(3p^{5}4f{\;}^{3}F_{3}\right)$, $|22\rangle :-0.6854\left(3p^{5}4f{\;}^{3}F_{2}\right)-0.5668\left(3p^{5}4f{\;}^{1}D_{2}*\right)-0.4568\left(3p^{5}4f{\;}^{3}D_{2}\right)$
35	No	No	$|10\rangle :-0.7312\left(3d^{9}4d{\;}^{3}F_{3}\right)+0.6100\left(3d^{9}4d{\;}^{1}F_{3}\right)-0.3018\left(3d^{9}4d{\;}^{3}D_{3}\right)$, $|11\rangle :0.7267\left(3d^{9}4d{\;}^{3}F_{2}\right)+0.6762\left(3d^{9}4d{\;}^{1}D_{2}\right)-0.1170\left(3d^{9}4d{\;}^{3}P_{2}\right)$
36	Yes	$\Delta E_{11,10}=0.00025$	$|10\rangle :0.7253\left(3d^{9}4d{\;}^{3}F_{2}\right)+0.6769\left(3d^{9}4d{\;}^{1}D_{2}\right)-0.1204\left(3d^{9}4d{\;}^{3}P_{2}\right)$, $|11\rangle :-0.7315\left(3d^{9}4d{\;}^{3}F_{3}\right)+0.6074\left(3d^{9}4d{\;}^{1}F_{3}\right)-0.3057\left(3d^{9}4d{\;}^{3}D_{3}\right)$
			$|18\rangle :0.7251\left(3p^{5}4f{\;}^{1}D_{2}\right)-0.6756\left(3p^{5}4f{\;}^{3}F_{2}\right)+0.1290\left(3p^{5}4f{\;}^{3}D_{2}\right)$, $|19\rangle :0.6373\left(3p^{5}4f{\;}^{1}F_{3}\right)+0.5552\left(3p^{5}4f{\;}^{3}F_{3}\right)-0.5342\left(3p^{5}4f{\;}^{3}G_{3}\right)$
37	Yes	$\Delta E_{19,18}=0.00049$	$|18\rangle :0.6363\left(3p^{5}4f{\;}^{1}F_{3}\right)+0.5534\left(3p^{5}4f{\;}^{3}F_{3}\right)-0.5373\left(3p^{5}4f{\;}^{3}G_{3}\right)$, $|19\rangle :0.7367\left(3p^{5}4f{\;}^{1}D_{2}\right)-0.6682\left(3p^{5}4f{\;}^{3}F_{2}\right)+0.0356\left(3s4d{\;}^{1}D_{2}\right)$
44	No	No	$|9\rangle :-0.6318\left(3d^{9}4d{\;}^{3}D_{2}\right)+0.4842\left(3d^{9}4d{\;}^{1}D_{2}\right)-0.4767\left(3d^{9}4d{\;}^{3}F_{2}\right)$,$|10\rangle :0.7672\left(3d^{9}4d{\;}^{3}F_{2}\right)+0.6257\left(3d^{9}4d{\;}^{1}D_{2}\right)-0.1390\left(3d^{9}4d{\;}^{3}P_{2}\right)$
45	Yes	$\Delta E_{10,9}=0.00075$	$|9\rangle :-0.7051\left(3d^{9}4d{\;}^{3}F_{2}\right)-0.6760\left(3d^{9}4d{\;}^{1}D_{2}\right)+0.1895\left(3d^{9}4d{\;}^{3}P_{2}\right)$,$|10\rangle :-0.6321\left(3d^{9}4d{\;}^{3}D_{2}\right)-0.5616\left(3d^{9}4d{\;}^{3}F_{2}\right)+0.3931\left(3d^{9}4d{\;}^{1}D_{2}\right)$
48	No	$\Delta E_{21,20}=0.0026$	$|20\rangle :-0.6155\left(3p^{5}4f{\;}^{3}D_{3}\right)+0.6027\left(3p^{5}4f{\;}^{1}F_{3}\right)+0.3895\left(3p^{5}4f{\;}^{3}G_{3}\right)-0.3182\left(3p^{5}4f{\;}^{3}F_{3}*\right)$, $|21\rangle :-0.7491\left(3p^{5}4f{\;}^{3}G_{3}\right)-0.5608\left(3p^{5}4f{\;}^{3}F_{3}\right)-0.3202\left(3p^{5}4f{\;}^{3}D_{3}\right)$
49	No	No	$|20\rangle :0.6178\left(3p^{5}4f{\;}^{1}F_{3}\right)-0.5314\left(3p^{5}4f{\;}^{3}D_{3}\right)+0.5453\left(3p^{5}4f{\;}^{3}G_{3}\right)-0.1865\left(3p^{5}4f{\;}^{3}F_{3}*\right)$, $|21\rangle :-0.6457\left(3p^{5}4f{\;}^{3}G_{3}\right)-0.6153\left(3p^{5}4f{\;}^{3}F_{3}\right)-0.4490\left(3p^{5}4f{\;}^{3}D_{3}\right)$
50	Yes	No	$|20\rangle :-0.6694\left(3p^{5}4f{\;}^{3}G_{3}\right)-0.6038\left(3p^{5}4f{\;}^{1}F_{3}\right)+0.4265\left(3p^{5}4f{\;}^{3}D_{3}\right)$, $|21\rangle :-0.6457\left(3p^{5}4f{\;}^{3}G_{3}\right)-0.6153\left(3p^{5}4f{\;}^{3}F_{3}\right)-0.4490\left(3p^{5}4f{\;}^{3}D_{3}\right)$
72	No	No	$|21\rangle :-0.7163\left(3p^{5}4f{\;}^{3}D_{3}\right)-0.5624\left(3p^{5}4f{\;}^{3}F_{3}\right)+0.3765\left(3p^{5}4f{\;}^{1}F_{3}\right)$, $|22\rangle :0.6647\left(3p^{5}4f{\;}^{3}F_{2}\right)+0.5863\left(3p^{5}4f{\;}^{1}D_{2}\right)+0.4473\left(3p^{5}4f{\;}^{3}D_{2}\right)$
73	Yes	$\Delta E_{22,21}=0.0021$	$|21\rangle :0.6645\left(3p^{5}4f{\;}^{3}F_{2}\right)+0.5858\left(3p^{5}4f{\;}^{1}D_{2}\right)+0.4480\left(3p^{5}4f{\;}^{3}D_{2}\right)$, $|22\rangle :0.7172\left(3p^{5}4f{\;}^{3}D_{3}\right)+0.5613\left(3p^{5}4f{\;}^{3}F_{3}\right)-0.3768\left(3p^{5}4f{\;}^{1}F_{3}\right)$
75	Yes	$\Delta E_{16,15}=0.1307$	$|15\rangle :0.5613\left(3p^{5}4f{\;}^{3}D_{2}\right)-0.5387\left(3p^{5}4p{\;}^{3}D_{2}\right)+0.4467\left(3p^{5}4p{\;}^{1}D_{2}*\right)$, $|16\rangle :-0.6291\left(3p^{5}4f{\;}^{3}D_{2}\right)+0.4665\left(3p^{5}4f{\;}^{3}F_{2}\right)-0.4393\left(3p^{5}4p{\;}^{3}D_{2}\right)$
		$\Delta E_{17,16}=0.1326$	$|17\rangle :0.7658\left(3p^{5}4f{\;}^{3}F_{3}\right)-0.4401\left(3p^{5}4f{\;}^{3}G_{3}\right)-0.3439\left(3p^{5}4f{\;}^{3}D_{3}\right)$, $|18\rangle :0.7425\left(3p^{5}4f{\;}^{1}F_{3}\right)+0.5870\left(3p^{5}4f{\;}^{3}D_{3}\right)-0.2581\left(3p^{5}4f{\;}^{3}G_{3}\right)$
76	Yes	$\Delta E_{18,17}=0.1121$	$|15\rangle :-0.8421\left(3p^{5}4f{\;}^{3}D_{2}\right)+0.5204\left(3p^{5}4f{\;}^{3}F_{2}\right)+0.0617\left(3p^{5}4f{\;}^{1}D_{2}\right)$, $|16\rangle :0.7619\left(3p^{5}4f{\;}^{3}F_{3}\right)-0.4451\left(3p^{5}4f{\;}^{3}G_{3}\right)+0.3340\left(3p^{5}4f{\;}^{1}F_{3}\right)$
		$\Delta E_{19,18}=0.2295$	$|17\rangle :0.7359\left(3p^{5}4f{\;}^{1}F_{3}\right)+0.5936\left(3p^{5}4f{\;}^{3}D_{3}\right)-0.2484\left(3p^{5}4f{\;}^{3}G_{3}\right)$, $|18\rangle :0.6036\left(3p^{5}4p{\;}^{3}D_{2}\right)-0.4937\left(3p^{5}4p{\;}^{1}D_{2}*\right)-0.4938\left(3p^{5}4p{\;}^{1}D_{2}\right)$, $|19\rangle :-0.6992\left(3p^{5}4f{\;}^{1}D_{2}\right)+0.4244\left(3p^{5}4f{\;}^{3}F_{2}\right)+0.3512\left(3p^{5}4p{\;}^{3}D_{2}\right)$
77	No	No	$|18\rangle :0.7867\left(3p^{5}4f{\;}^{1}D_{2}\right)-0.5112\left(3p^{5}4f{\;}^{3}F_{2}\right)-0.2660\left(3p^{5}4f{\;}^{3}D_{2}\right)$, $|19\rangle :0.6852\left(3p^{5}4p{\;}^{3}D_{2}\right)-0.5638\left(3p^{5}4p{\;}^{1}D_{2}*\right)+0.3998\left(3p^{5}4p{\;}^{3}P_{2}\right)$
86	Yes	No	$|10\rangle :-0.6814\left(3d^{9}4d{\;}^{3}D_{2}\right)-0.4893\left(3d^{9}4d{\;}^{3}P_{2}*\right)+0.3657\left(3d^{9}4d{\;}^{1}D_{2}\right)$, $|11\rangle :0.7121\left(3d^{9}4d{\;}^{3}F_{3}\right)-0.5042\left(3d^{9}4d{\;}^{1}F_{3}\right)+0.4197\left(3d^{9}4d{\;}^{3}D_{3}\right)$, $|12\rangle :-0.6912\left(3p^{5}4p{\;}^{3}D_{2}\right)-0.5647\left(3p^{5}4p{\;}^{1}D_{2}*\right)+0.3972\left(3p^{5}4p{\;}^{3}P_{2}\right)$
87	Yes	$\Delta E_{12,11}=0.0484$	$|10\rangle :-0.6320\left(3p^{5}4p{\;}^{3}D_{2}\right)-0.5136\left(3p^{5}4p{\;}^{1}D_{2}*\right)+0.3492\left(3p^{5}4p{\;}^{3}P_{2}\right)$, $|11\rangle :-0.6463\left(3d^{9}4d{\;}^{3}D_{2}\right)-0.4138\left(3d^{9}4d{\;}^{3}P_{2}*\right)-0.3501\left(3d^{9}4d{\;}^{3}F_{2}\right)$, $|12\rangle :0.7119\left(3d^{9}4d{\;}^{3}F_{3}\right)-0.5037\left(3d^{9}4d{\;}^{1}F_{3}\right)+0.4199\left(3d^{9}4d{\;}^{3}D_{3}\right)$
90	No	No	$|9\rangle :-0.7679\left(3d^{9}4d{\;}^{3}F_{2}\right)-0.4893\left(3d^{9}4d{\;}^{1}D_{2}\right)-0.2641\left(3p^{5}4p{\;}^{3}D_{2}\right)$, $|10\rangle :-0.6552\left(3p^{5}4p{\;}^{3}D_{2}\right)-0.5333\left(3p^{5}4p{\;}^{1}D_{2}*\right)+0.3714\left(3p^{5}4p{\;}^{3}P_{2}\right)$
91	Yes	$\Delta E_{9,8}=0.0073$	$|8\rangle :0.9084\left(3d^{9}4d{\;}^{3}G_{3}\right)$, $|9\rangle :-0.6139\left(3p^{5}4p{\;}^{3}D_{2}\right)-0.5030\left(3p^{5}4p{\;}^{1}D_{2}*\right)-0.4321\left(3d^{9}4d{\;}^{3}F_{2}\right)$, $|10\rangle :-0.6901\left(3d^{9}4d{\;}^{3}F_{2}\right)-0.4892\left(3d^{9}4d{\;}^{1}D_{2}\right)+0.3496\left(3p^{5}4p{\;}^{3}D_{2}\right)$
92	No	No	$|8\rangle :-0.6908\left(3p^{5}4p{\;}^{3}D_{2}\right)-0.5648\left(3p^{5}4p{\;}^{1}D_{2}*\right)+0.3941\left(3p^{5}4p{\;}^{3}P_{2}\right)$, $|9\rangle :0.9084\left(3d^{9}4d{\;}^{3}G_{3}\right)$

**Table 10:** Sudden change of Shannon entropies, eigenlevel anticrossings (in a.u.), configuration mixing coefficients, and information exchanges for the levels in the LS-coupled subspace with $J^{P}=4^{e}$ and 5^*e*^.

Z	Sudden Change	Eigenlevel Anticrossing	Configuration Mixing Coefficients
35	No	No	$|1\rangle :0.9999\left(3d^{9}4d{\;}^{3}G_{5}\right)$, $|2\rangle :0.7968\left(3d^{9}4d{\;}^{3}G_{4}\right)+0.5952\left(3d^{9}4d{\;}^{1}G_{4}\right)-0.1029\left(3d^{9}4d{\;}^{3}F_{4}\right)$
36	Yes	$\Delta E_{2,1}=6.0\times 10^{-5}$	$|1\rangle :0.7940\left(3d^{9}4d{\;}^{3}G_{4}\right)+0.5980\left(3d^{9}4d{\;}^{1}G_{4}\right)-0.1086\left(3d^{9}4d{\;}^{3}F_{4}\right)$, $|2\rangle :0.9999\left(3d^{9}4d{\;}^{3}G_{5}\right)$
61	No	$\Delta E_{6,5}=3.0\times 10^{-5}$	$|5\rangle :0.9998\left(3p^{5}4f{\;}^{3}G_{5}\right)$, $|6\rangle :0.7470\left(3p^{5}4f{\;}^{3}G_{4}\right)+0.6330\left(3p^{5}4f{\;}^{1}G_{4}\right)-0.2025\left(3p^{5}4f{\;}^{3}F_{4}\right)$
62	Yes	No	$|5\rangle :0.7467\left(3p^{5}4f{\;}^{3}G_{4}\right)+0.6332\left(3p^{5}4f{\;}^{1}G_{4}\right)-0.2032\left(3p^{5}4f{\;}^{3}F_{4}\right)$, $|6\rangle :0.9999\left(3p^{5}4f{\;}^{3}G_{5}\right)$

**Table 11:** Sudden change of Shannon entropies, eigenlevel anticrossings (in a.u.), configuration mixing coefficients, and information exchanges for the levels in the LS-coupled subspace with $J^{P}=0^{o}$.

Z	Sudden Change	Eigenlevel Anticrossing	Configuration Mixing Coefficients
77	No	No	$|3\rangle :0.8557\left(3p^{5}4s{\;}^{3}P_{0}\right)-0.5126\left(3p^{5}4d{\;}^{3}P_{0}\right)$, $|4\rangle :0.8559\left(3p^{5}4d{\;}^{3}P_{0}\right)+0.5153\left(3p^{5}4s{\;}^{3}P_{0}\right)$
78	Yes	$\Delta E_{4,3}=0.5284$	$|3\rangle :0.7783\left(3p^{5}4d{\;}^{3}P_{0}\right)-0.6231\left(3p^{5}4s{\;}^{3}P_{0}\right)$, $|4\rangle :0.7807\left(3p^{5}4s{\;}^{3}P_{0}\right)-0.6242\left(3p^{5}4d{\;}^{3}P_{0}\right)$

**Table 12:** Sudden change of Shannon entropies, eigenlevel anticrossings (in a.u.), configuration mixing coefficients, and information exchanges for the levels in the LS-coupled subspace with $J^{P}=1^{o}$.

Z	Sudden Change	Eigenlevel Anticrossing	Configuration Mixing Coefficients
49	Yes	$\Delta E_{7,6}=0.11137$	$|6\rangle :-0.8459\left(3d^{9}4f{\;}^{1}P_{1}\right)+0.4001\left(3p^{5}4s{\;}^{1}P_{1}\right)+0.2725\left(3p^{5}4s{\;}^{3}P_{1}\right)$, $|7\rangle :0.6894\left(3p^{5}4s{\;}^{1}P_{1}\right)+0.5366\left(3p^{5}4s{\;}^{3}P_{1}\right)+0.4689\left(3d^{9}4f{\;}^{1}P_{1}\right)$
50	Yes	No	$|6\rangle :0.7357\left(3p^{5}4s{\;}^{1}P_{1}\right)+0.5383\left(3p^{5}4s{\;}^{3}P_{1}\right)-0.3928\left(3d^{9}4f{\;}^{1}P_{1}\right)$, $|7\rangle :0.8819\left(3d^{9}4f{\;}^{1}P_{1}\right)+0.3097\left(3p^{5}4s{\;}^{1}P_{1}\right)+0.2658\left(3p^{5}4s{\;}^{3}P_{1}\right)$
55	Yes	$\Delta E_{6,5}=0.01691$	$|5\rangle :-0.6319\left(3d^{9}4f{\;}^{3}D_{1}\right)-0.5370\left(3p^{5}4s{\;}^{1}P_{1}\right)-0.3847\left(3p^{5}4s{\;}^{3}P_{1}\right)$, $|6\rangle :-0.6149\left(3d^{9}4f{\;}^{3}D_{1}\right)+0.5948\left(3p^{5}4s{\;}^{1}P_{1}*\right)+0.4461\left(3p^{5}4s{\;}^{3}P_{1}\right)$
56	No	No	$|5\rangle :0.7995\left(3p^{5}4s{\;}^{1}P_{1}\right)+0.5853\left(3p^{5}4s{\;}^{3}P_{1}\right)-0.0874\left(3d^{9}4f{\;}^{1}P_{1}\right)$, $|6\rangle :-0.8760\left(3d^{9}4f{\;}^{3}D_{1}\right)-0.3710\left(3d^{9}4f{\;}^{3}P_{1}\right)+0.2959\left(3d^{9}4f{\;}^{1}P_{1}\right)$
58	No	No	$|4\rangle :0.8778\left(3d^{9}4f{\;}^{3}P_{1}\right)-0.4081\left(3d^{9}4f{\;}^{3}D_{1}\right)-0.1928\left(3p^{5}4s{\;}^{1}P_{1}\right)$, $|5\rangle :0.7813\left(3p^{5}4s{\;}^{1}P_{1}\right)+0.5686\left(3p^{5}4s{\;}^{3}P_{1}\right)+0.2271\left(3d^{9}4f{\;}^{3}P_{1}\right)$
59	Yes	$\Delta E_{5,4}=0.05159$	$|4\rangle :0.7126\left(3p^{5}4s{\;}^{1}P_{1}\right)+0.5247\left(3p^{5}4s{\;}^{3}P_{1}\right)-0.4141\left(3d^{9}4f{\;}^{3}P_{1}\right)$, $|5\rangle :0.8031\left(3d^{9}4f{\;}^{3}P_{1}\right)+0.3757\left(3p^{5}4s{\;}^{1}P_{1}\right)-0.3721\left(3d^{9}4f{\;}^{3}D_{1}\right)$
71	Yes	$\Delta E_{12,11}=0.6555$	$|11\rangle :0.5798\left(3s4p{\;}^{3}P_{1}\right)+0.5195\left(3p^{5}4d{\;}^{3}D_{1}*\right)+0.4108\left(3s4p{\;}^{1}P_{1}\right)$, $|12\rangle :-0.5895\left(3s4p{\;}^{3}P_{1}\right)+0.4898\left(3p^{5}4d{\;}^{3}D_{1}\right)+0.4141\left(3p^{5}4d{\;}^{1}P_{1}\right)$
72	No	No	$|11\rangle :0.6459\left(3s4p{\;}^{3}P_{1}\right)+0.4563\left(3p^{5}4d{\;}^{3}D_{1}\right)+0.4540\left(3s4p{\;}^{1}P_{1}\right)$, $|12\rangle :0.5487\left(3p^{5}4d{\;}^{3}D_{1}\right)-0.5155\left(3s4p{\;}^{3}P_{1}\right)+0.4591\left(3p^{5}4d{\;}^{1}P_{1}\right)$
77	No	No	$|8\rangle :0.7658\left(3p^{5}4d{\;}^{3}P_{1}\right)-0.5392\left(3p^{5}4d{\;}^{3}D_{1}\right)-0.2530\left(3p^{5}4s{\;}^{3}P_{1}\right)$, $|9\rangle :0.7611\left(3p^{5}4s{\;}^{3}P_{1}\right)-0.5425\left(3p^{5}4s{\;}^{1}P_{1}\right)-0.2490\left(3p^{5}4d{\;}^{3}D_{1}\right)$
78	Yes	$\Delta E_{9,8}=0.3099$	$|8\rangle :0.5958\left(3p^{5}4d{\;}^{3}P_{1}\right)-0.5647\left(3p^{5}4s{\;}^{3}P_{1}\right)+0.4131\left(3p^{5}4s{\;}^{1}P_{1}\right)$, $|9\rangle :-0.5764\left(3p^{5}4s{\;}^{3}P_{1}\right)-0.5193\left(3p^{5}4d{\;}^{3}P_{1}\right)+0.4059\left(3p^{5}4s{\;}^{1}P_{1}\right)$
81	Yes	$\Delta E_{10,9}=0.2760$	$|9\rangle :0.6168\left(3p^{5}4s{\;}^{3}P_{1}\right)+0.5274\left(3p^{5}4d{\;}^{1}P_{1}\right)-0.4421\left(3p^{5}4s{\;}^{1}P_{1}\right)$, $|10\rangle :-0.6001\left(3p^{5}4d{\;}^{1}P_{1}\right)-0.5210\left(3p^{5}4s{\;}^{3}P_{1}\right)-0.4081\left(3p^{5}4d{\;}^{3}P_{1}\right)$
82	No	No	$|9\rangle :0.7611\left(3p^{5}4d{\;}^{1}P_{1}\right)-0.4172\left(3p^{5}4d{\;}^{3}P_{1}\right)-0.3845\left(3p^{5}4d{\;}^{3}D_{1}\right)$, $|10\rangle :0.7695\left(3p^{5}4s{\;}^{3}P_{1}\right)-0.5492\left(3p^{5}4s{\;}^{1}P_{1}\right)-0.2383\left(3p^{5}4d{\;}^{1}P_{1}\right)$

**Table 13:** Sudden change of Shannon entropies, eigenlevel anticrossings (in a.u.), configuration mixing coefficients, and information exchanges for the levels in the LS-coupled subspace with $J^{P}=2^{o}$.

Z	Sudden Change	Eigenlevel Anticrossing	Configuration Mixing Coefficients
52	Yes	$\Delta E_{9,8}=0.02944$	$|7\rangle :-0.6123\left(3d^{9}4f{\;}^{3}P_{2}\right)-0.5682\left(3d^{9}4f{\;}^{3}D_{2}*\right)+0.5127\left(3d^{9}4f{\;}^{1}D_{2}\right)$, $|8\rangle :0.7880\left(3d^{9}4f{\;}^{3}F_{2}\right)+0.4498\left(3d^{9}4f{\;}^{1}D_{2}\right)+0.4166\left(3d^{9}4f{\;}^{3}D_{2}\right)$, $|9\rangle :-0.9773\left(3p^{5}4s{\;}^{3}P_{2}\right)$
53	Yes	$\Delta E_{8,7}=0.05352$	$|7\rangle :-0.8822\left(3p^{5}4s{\;}^{3}P_{2}\right)+0.2936\left(3d^{9}4f{\;}^{3}D_{2}\right)-0.2600\left(3d^{9}4f{\;}^{1}D_{2}\right)$, $|8\rangle :-0.5882\left(3d^{9}4f{\;}^{3}P_{2}\right)-0.4620\left(3p^{5}4s{\;}^{3}P_{2}\right)-0.4815\left(3d^{9}4f{\;}^{3}D_{2}*\right)$, $|9\rangle :0.7865\left(3d^{9}4f{\;}^{3}F_{2}\right)+0.4403\left(3d^{9}4f{\;}^{1}D_{2}\right)+0.4294\left(3d^{9}4f{\;}^{3}D_{2}\right)$
56	No	No	$|5\rangle :-0.7305\left(3d^{9}4f{\;}^{3}P_{2}\right)+0.5644\left(3d^{9}4f{\;}^{3}D_{2}\right)-0.3171\left(3d^{9}4f{\;}^{1}D_{2}\right)$, $|6\rangle :-0.6507\left(3d^{9}4f{\;}^{1}D_{2}\right)+0.6110\left(3d^{9}4f{\;}^{3}F_{2}\right)-0.4310\left(3d^{9}4f{\;}^{3}D_{2}\right)$, $|7\rangle :-0.9740\left(3p^{5}4s{\;}^{3}P_{2}\right)$, $|8\rangle :0.6597\left(3d^{9}4f{\;}^{3}P_{2}\right)+0.5330\left(3d^{9}4f{\;}^{3}D_{2}*\right)-0.5211\left(3d^{9}4f{\;}^{1}D_{2}\right)$
57	Yes	$\Delta E_{6,5}=0.0630$	$|5\rangle :-0.6371\left(3d^{9}4f{\;}^{3}P_{2}\right)+0.4874\left(3d^{9}4f{\;}^{3}D_{2}*\right)+0.5234\left(3p^{5}4s{\;}^{3}P_{2}\right)$, $|6\rangle :-0.8445\left(3p^{5}4s{\;}^{3}P_{2}\right)+0.3667\left(3d^{9}4f{\;}^{3}D_{2}\right)-0.3514\left(3d^{9}4f{\;}^{3}P_{2}\right)$
		$\Delta E_{7,6}=0.0486$	$|7\rangle :0.6767\left(3d^{9}4f{\;}^{1}D_{2}\right)+0.3833\left(3d^{9}4f{\;}^{3}D_{2}\right)-0.6046\left(3d^{9}4f{\;}^{3}F_{2}\right)$, $|8\rangle :-0.6644\left(3d^{9}4f{\;}^{3}P_{2}\right)+0.5236\left(3d^{9}4f{\;}^{3}D_{2}\right)-0.5239\left(3d^{9}4f{\;}^{1}D_{2}\right)$
58	Yes	No	$|5\rangle :-0.9156\left(3p^{5}4s{\;}^{3}P_{2}\right)$, $|6\rangle :-0.6810\left(3d^{9}4f{\;}^{3}P_{2}\right)+0.5946\left(3d^{9}4f{\;}^{3}D_{2}\right)-0.2973\left(3p^{5}4s{\;}^{3}P_{2}\right)$, $|7\rangle :0.6797\left(3d^{9}4f{\;}^{1}D_{2}\right)-0.6067\left(3d^{9}4f{\;}^{3}F_{2}\right)+0.3811\left(3d^{9}4f{\;}^{3}D_{2}\right)$, $|8\rangle :-0.6686\left(3d^{9}4f{\;}^{3}P_{2}\right)+0.5272\left(3d^{9}4f{\;}^{1}D_{2}\right)-0.5132\left(3p^{5}4s{\;}^{3}D_{2}*\right)$

**Table 14:** Sudden change of Shannon entropies, eigenlevel anticrossings (in a.u.), configuration mixing coefficients, and information exchanges for the levels in the LS-coupled subspace with $J^{P}=3^{o}$ and $4^{o}$.

Z	Sudden Change	Eigenlevel Anticrossing	Configuration Mixing Coefficients
35	No	No	$|13\rangle :0.9996\left(3p^{5}4d{\;}^{3}F_{4}\right)$, $|14\rangle :0.7238\left(3p^{5}4d{\;}^{3}F_{3}\right)+0.6750\left(3p^{5}4d{\;}^{1}F_{3}\right)-0.1405\left(3p^{5}4d{\;}^{3}D_{3}\right)$
36	Yes	$\Delta E_{14,13}=0.00023$	$|13\rangle :0.7238\left(3p^{5}4d{\;}^{3}F_{3}\right)+0.6745\left(3p^{5}4d{\;}^{1}F_{3}\right)-0.1418\left(3p^{5}4d{\;}^{3}D_{3}\right)$, $|14\rangle :0.9995\left(3p^{5}4d{\;}^{3}F_{4}\right)$
39	No	$\Delta E_{18,17}=4.0\times 10^{-5}$	$|17\rangle :-0.9998\left(3s4f{\;}^{3}F_{4}\right)$, $|18\rangle :-0.9998\left(3s4f{\;}^{3}F_{3}\right)$
40	Yes	No	$|17\rangle :-0.9997\left(3s4f{\;}^{3}F_{3}\right)$, $|18\rangle :-0.9998\left(3s4f{\;}^{3}F_{4}\right)$
56	No	$\Delta E_{12,11}=2.0\times 10^{-5}$	$|11\rangle :-0.6741\left(3d^{9}4f{\;}^{3}G_{4}\right)+0.5781\left(3d^{9}4f{\;}^{1}G_{4}\right)-0.4577\left(3d^{9}4f{\;}^{3}F_{4}\right)$, $|12\rangle :-0.7050\left(3d^{9}4f{\;}^{3}G_{3}\right)-0.6430\left(3d^{9}4f{\;}^{1}F_{3}\right)+0.2952\left(3d^{9}4f{\;}^{3}D_{3}\right)$
57	Yes	No	$|11\rangle :-0.6919\left(3d^{9}4f{\;}^{3}G_{3}\right)-0.6483\left(3d^{9}4f{\;}^{1}F_{3}\right)+0.3109\left(3d^{9}4f{\;}^{3}D_{3}\right)$, $|12\rangle :-0.6742\left(3d^{9}4f{\;}^{3}G_{4}\right)+0.5762\left(3d^{9}4f{\;}^{1}G_{4}\right)-0.4596\left(3d^{9}4f{\;}^{3}F_{4}\right)$
60	No		$|10\rangle :0.6384\left(3d^{9}4f{\;}^{3}F_{3}\right)+0.6009\left(3d^{9}4f{\;}^{3}G_{3}\right)+0.4524\left(3d^{9}4f{\;}^{3}D_{3}*\right)$, $|11\rangle :-0.6691\left(3d^{9}4f{\;}^{1}F_{3}\right)-0.6224\left(3d^{9}4f{\;}^{3}G_{3}*\right)+0.3780\left(3d^{9}4f{\;}^{3}D_{3}\right)$
61	No		$|10\rangle :0.6403\left(3d^{9}4f{\;}^{3}G_{3}\right)+0.6279\left(3d^{9}4f{\;}^{3}F_{3}\right)+0.4269\left(3d^{9}4f{\;}^{3}D_{3}\right)$, $|11\rangle :-0.6764\left(3d^{9}4f{\;}^{1}F_{3}\right)-0.5816\left(3d^{9}4f{\;}^{3}G_{3}\right)+0.4096\left(3d^{9}4f{\;}^{3}D_{3}*\right)$
62	No		$|10\rangle :0.6861\left(3d^{9}4f{\;}^{3}G_{3}\right)+0.6103\left(3d^{9}4f{\;}^{3}F_{3}\right)+0.3915\left(3d^{9}4f{\;}^{3}D_{3}\right)$, $|11\rangle :-0.6815\left(3d^{9}4f{\;}^{1}F_{3}\right)-0.5266\left(3d^{9}4f{\;}^{3}G_{3}\right)+0.4461\left(3d^{9}4f{\;}^{3}D_{3}*\right)$
63	No	$\Delta E_{11,10}=0.0274$	$|10\rangle :0.7356\left(3d^{9}4f{\;}^{3}G_{3}\right)+0.5830\left(3d^{9}4f{\;}^{3}F_{3}\right)+0.3443\left(3d^{9}4f{\;}^{3}D_{3}\right)$, $|11\rangle :-0.6815\left(3d^{9}4f{\;}^{1}F_{3}\right)+0.4857\left(3d^{9}4f{\;}^{3}D_{3}*\right)-0.4548\left(3d^{9}4f{\;}^{3}G_{3}\right)$
64	Yes	No	$|10\rangle :0.7824\left(3d^{9}4f{\;}^{3}G_{3}\right)+0.5448\left(3d^{9}4f{\;}^{3}F_{3}\right)+0.2864\left(3d^{9}4f{\;}^{3}D_{3}\right)$, $|11\rangle :-0.6730\left(3d^{9}4f{\;}^{1}F_{3}\right)+0.5240\left(3d^{9}4f{\;}^{3}D_{3}*\right)+0.3696\left(3d^{9}4f{\;}^{3}G_{3}\right)$

**Table 15:** Sudden change of Shannon entropies, eigenlevel anticrossings (in a.u.), configuration mixing coefficients, and information exchanges for the levels in the LS-coupled subspace with $J^{P}=5^{o}$ and $6^{o}$.

Z	Sudden Change	Eigenlevel Anticrossing	Configuration Mixing Coefficients
56	Yes	$\Delta E_{2,1}=0.00028$	$|1\rangle :\left(3d^{9}4f{\;}^{3}H_{6}\right)$, $|2\rangle :0.7408\left(3d^{9}4f{\;}^{3}H_{5}\right)+0.6615\left(3d^{9}4f{\;}^{1}H_{5}\right)-0.1173\left(3d^{9}4f{\;}^{3}G_{5}\right)$
57	No	No	$|1\rangle :0.7396\left(3d^{9}4f{\;}^{3}H_{5}\right)+0.6622\left(3d^{9}4f{\;}^{1}H_{5}\right)-0.1205\left(3d^{9}4f{\;}^{3}G_{5}\right)$, $|2\rangle :\left(3d^{9}4f{\;}^{3}H_{6}\right)$
